# Petasis vs. Strecker Amino Acid Synthesis: Convergence, Divergence and Opportunities in Organic Synthesis

**DOI:** 10.3390/molecules26061707

**Published:** 2021-03-18

**Authors:** Wayiza Masamba

**Affiliations:** Department of Chemical and Physical Sciences, Faculty of Natural Sciences, Walter Sisulu University, Nelson Mandela Drive, Mthatha 5117, South Africa; wmasambal@wsu.ac.za

**Keywords:** amino acids, biomolecules, multicomponent reactions, peptides, Petasis, Strecker

## Abstract

α-Amino acids find widespread applications in various areas of life and physical sciences. Their syntheses are carried out by a multitude of protocols, of which Petasis and Strecker reactions have emerged as the most straightforward and most widely used. Both reactions are three-component reactions using the same starting materials, except the nucleophilic species. The differences and similarities between these two important reactions are highlighted in this review.

## 1. Introduction

The inadvertent discovery of the synthesis of α-amino acids by Strecker in 1850, in an attempt to prepare lactic acid from a mixture of ammonia, acetaldehyde and hydrogen cyanide [1,2,3], marked the dawn of new and stimulating advances in physical and life sciences. Indeed, following this discovery, α-amino acids, the “building blocks of life”, became readily accessible, pushing back the boundaries in peptide and protein research. This reaction, known as the “Strecker synthesis”, belongs to the general group of multicomponent reactions, and quickly became a powerful tool in the preparation of both biogenic and non-natural α-amino acids and associated substances, such as hydantoins [4,5] and polypeptides. As expected of most multicomponent reactions, the Strecker reaction allows the rapid and efficient assembly of complex α-amino acid derivatives whose preparation would otherwise be difficult, if not impossible by other methods. The simplicity of the reaction protocol as well as the possible availability of starting materials under prebiotic conditions strongly suggest that the Strecker amino acid synthesis is likely involved in the origin of life [6]. Therefore, this reaction prominently features in all discussions regarding the onset of life on earth.

α-Amino acids play a critical role in human nutrition and are extensively used as food additives thanks to their biological activity, as agrochemicals and as pharmaceuticals as well as a source for chiral building blocks in asymmetric syntheses of simple and more complex drug molecules [7,8].

**Figure 1:**
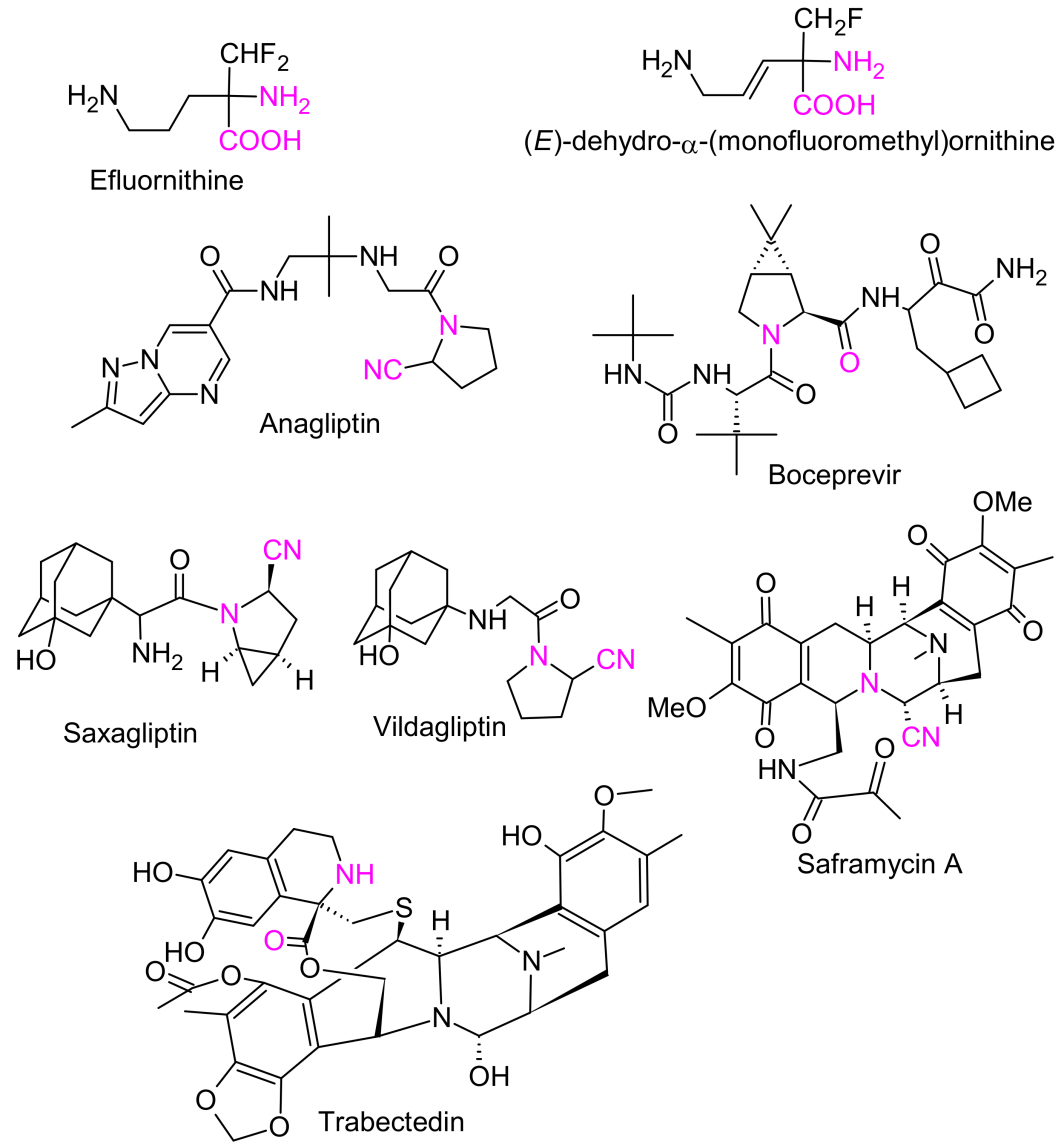
Examples of bioactive amino acids prepared by the Strecker synthesis. Purple colour shows the amino nitrile functions obtained as a result of a direct Strecker reaction.

In peptide and peptomimetics research, sterically hindered and α,α-disubstituted amino acids in particular have been used in numerous investigations because they induce restricted conformations when incorporated into a peptide, resulting in well-defined and pre-defined secondary structures and new properties [9]. This has guided the design of tailor-made amino acids whose integration into oligopeptides has allowed the identification of biologically active compounds such as the potent hepatitis C virus (HCV) NS3 serine protease inhibitor boceprevir [10] (see Figure 1).

Owing to all of the above reasons, the classical Strecker synthesis has gained popularity among organic chemists and remains to date the most economical and reliable method for the ready access to both natural and non-coded amino acids, and the most effective way for the preparation of large libraries of structurally diverse and distinct molecules.

**Scheme 1:**
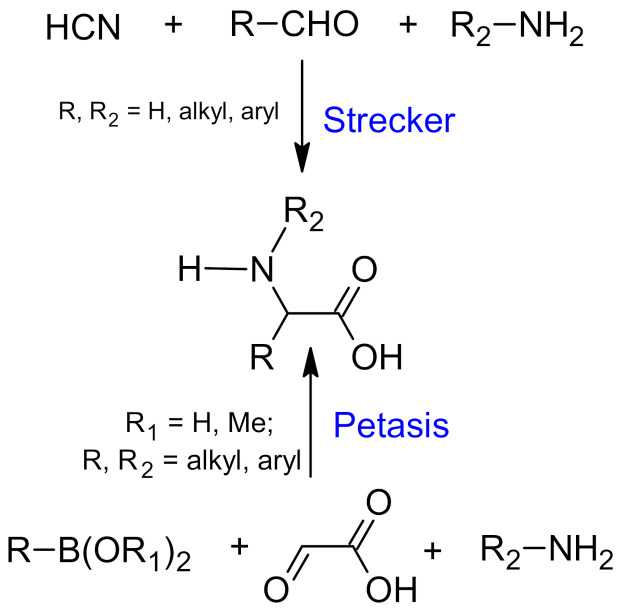
General Petasis and Strecker amino acid synthesis.

The Petasis reaction, on the other hand, is relatively new, appearing in the chemistry literature in 1993, more than a century later, when Petasis and his group reported, in its original version, the reaction of a secondary amine, paraformaldehyde, and (*E*)-vinylboronic acid to afford the corresponding allylamines in high yields [11]. Subsequent use of glyoxylic acid as the aldehyde component resulted in the formation of the corresponding α-amino acids, paving the way for another method for the synthesis of these molecules. Like the Strecker amino acid synthesis, this reaction has all the hallmarks of all multicomponent reactions: it involves the reaction of three starting materials in a one-pot fashion to afford more complex molecules containing most, if not all the reacting components. In addition, the reaction is characterised by its simplicity, is experimentally convenient and does not require anhydrous conditions or inert atmosphere. Scheme 1 summarizes the general features of these two reactions leading to the same reaction products.

There is an abundant literature regarding each of these two reactions and since they lead to the same important group of compounds, it appears relevant to compare and contrast both reactions, and outline their respective points of convergence and divergence, and delineate areas of complementarity. This is the goal we tried to achieve in this review, starting with a brief survey of each reaction and their recent developments.

## 2. Strecker Reaction

**Scheme 2:**
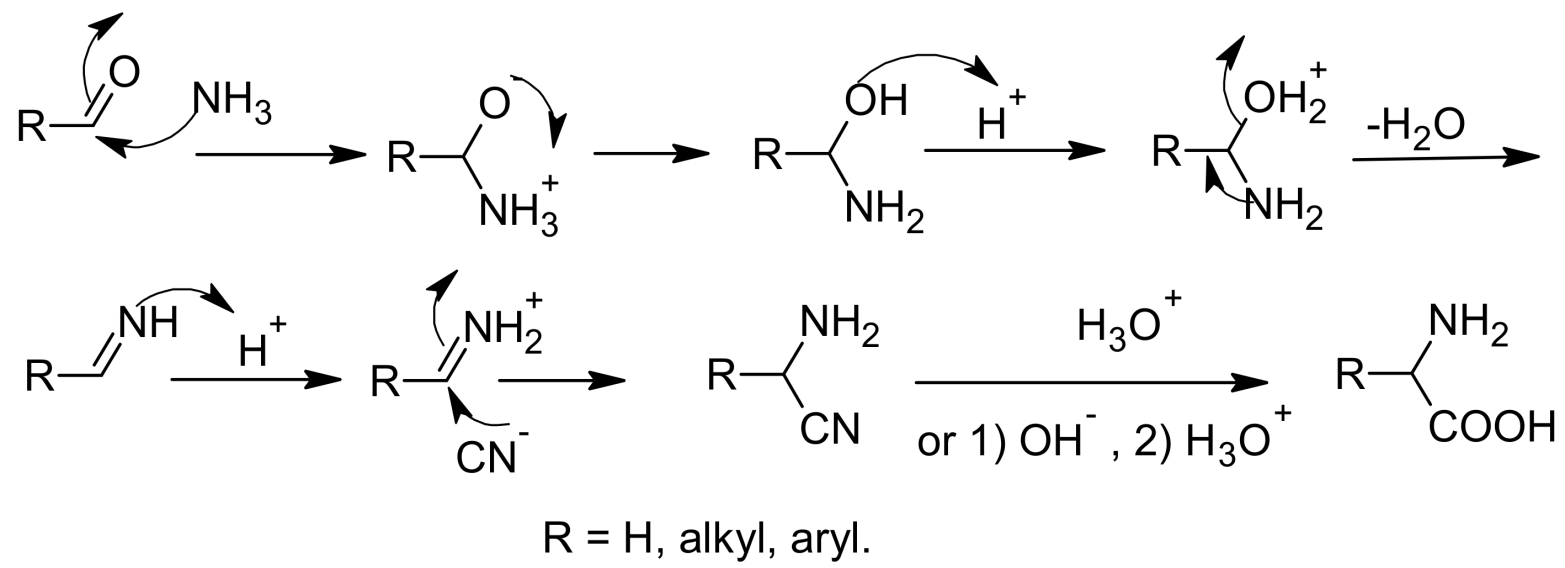
General mechanism of the Strecker amino acid synthesis.

The classical Strecker amino acid synthesis outlined in Scheme 1 quickly developed into an industrial synthesis of both natural and non-natural racemic α-amino acids. The mechanism of the reaction is widely accepted to start by the formation of the iminium ion by the addition of ammonia (or amine) to the aldehyde or ketone; this is followed by the nucleophilic attack of the cyanide, leading to the formation of the corresponding α-aminonitrile, whose subsequent hydrolysis under basic or acidic conditions forms the amino acid (Scheme 2).

In practice and for safety purposes, solid ammonium chloride is used as the ammonia source while gaseous hydrogen cyanide is replaced by solid sodium cyanide, which is more convenient to handle.

Compared to aldehydes, the reaction with ketones is sluggish due to the additional steric hindrance brought about by the substituent replacing the hydrogen atom [12]. Products are generally obtained as a racemic mixture; nevertheless, the method remains the tool of choice in the rapid assembly of large libraries of achiral amino nitriles, whose subsequent chemical modifications and resolution produce the desired amino acids in optically pure form.

Figure 1 outlines some of the biologically relevant amino acids prepared by the direct three-component Strecker synthesis, including both conformationally rigid α- and β-diamino acids [13], reflecting the diversity and complexity of molecular entities that can be rapidly constructed by this reaction. Eflornithine and (*E*)-dehydro-α-monofluoromethyl)ornithine are both irreversible inhibitors of ornithine decarboxylase [14], while trabectedin is an approved drug for the treatment of cancer [15]. Saframycin A, a potent antitumor alkaloid with a pentacyclic structure, was prepared by a single step involving a Strecker reaction of three key precursors [16]; anagliptin is known to decrease the cholesterol synthesis marker lathosterol [17] whereas vildagliptin and saxagliptin, are dipeptidyl peptidase IV (DPP-IV) inhibitors [18]. 

**Scheme 3:**
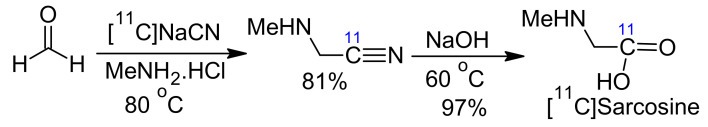
Synthesis of radiolabelled [^11^C]sarcosine.

The operational simplicity of the procedure has been exploited in automated processes for the synthesis of unusual amino acids such as radiolabelled [^11^C-carbonyl]sarcosine used in positron emission tomography (PET) imaging, starting from [^11^C]cyanide [19] (Scheme 3).

Beside [^11^C]sarcosine, the protocol was successfully applied in the production of a number of new PET radiotracers such as [^11^C-carbonyl]methionine, [^11^C-carbonyl]-*N*-phenylglycine and [^11^C-carbonyl]glycine in moderate to good radiochemical yields (79% overall yield).

Compared to earlier procedures based on the fixation of [^11^C]CO_2_ as the carbonyl source, using Grignard or organolithium reagents, the above method is clearly more straightforward and robust, whose sole limitation is the availability of the labelled cyanide.

A similar process to prepare d-[1-^14^C]-serine in high enantiomeric purity was employed, starting from [^14^C]-KCN using (*R*)-1-phenylethylamine as the chiral auxiliary [20].

Furthermore, membrane-active peptides can be studied under their natural conditions in lipid bilayers by solid-state NMR spectroscopy using ^19^F as the probe. Towards this end, Mykhailiuk et al. [21] carried out the Strecker reaction for the synthesis of the corresponding novel aliphatic ^19^F-substituted amino acids, which can be incorporated into bio-compatible peptides for their subsequent study by ^19^F-NMR spectroscopy.

The above few specific examples sufficiently demonstrate the importance of the Strecker reaction as a versatile tool in organic synthesis.

## 3. Green Strecker Reaction

The source of the cyanide in the Strecker synthesis varies considerably depending on the methods: certain protocols use HCN, KCN, TMSCN, (EtO)_2_P(O)CN, Et_2_AlCN, Bu_3_SnCN, MeCOCN, acetone cyanohydrin and ethyl cyanoformate [22], which are all toxic reagents.

As a multicomponent reaction, the Strecker synthesis already incorporates many aspects of green chemistry. Ironically, despite its widespread applications both in academia and industry, the use of highly toxic cyanides as one of the components, goes against one of the very principles of green chemistry [23], and poses serious risks to human health and the environment. In order to address this fundamental concern, many research efforts have been directed towards the replacement of the cyanide species by safer and more environmentally friendly alternatives.

Hexacyanoferrates are appealing as safe cyanide sources. Indeed, ferro- and ferricyanides are stable, inexpensive, and essentially non-toxic (their oral LD50 values in the rat are even higher than the corresponding value for NaCl). Despite their stability, they are known to release cyanide under a variety of conditions [24].

**Scheme 4:**
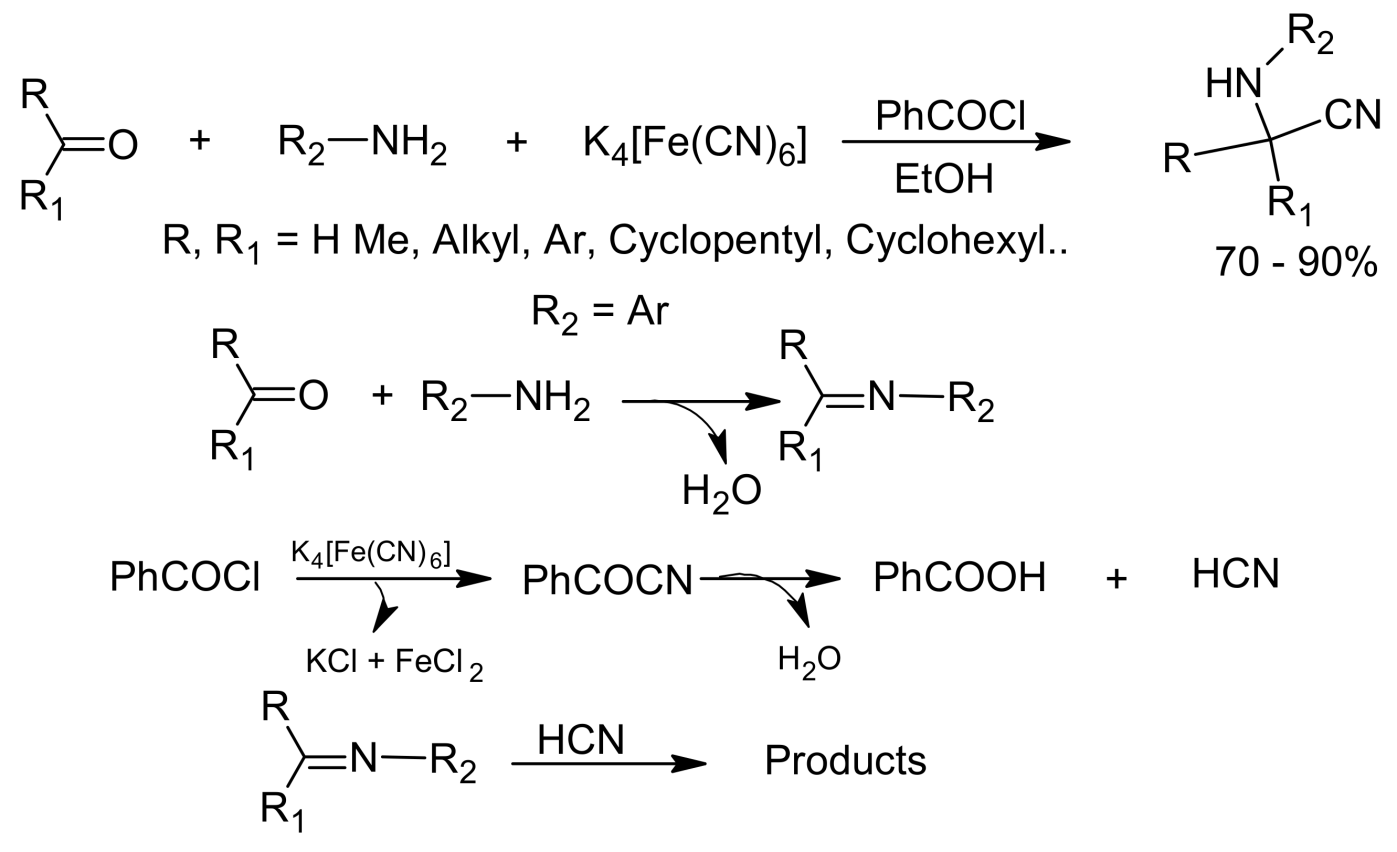
Green synthesis of α-amino nitriles in the presence of potassium ferrocyanide and its mechanism.

In 2010, the group of Li reported the first efficient and environmentally friendly synthesis of α-aminonitriles via one-pot three-component condensation of carbonyl compounds, amines, and potassium hexacyanoferrate (II) in the presence of benzoyl chloride as a promoter [22]. In this approach, the role of the promoter is to react with K_4_[Fe(CN)_6_] and generate benzoyl cyanide, whose reaction with water constitutes an in-situ source of HCN. Finally, nucleophilic addition of cyanide ions to the imines, which are formed from condensation of aldehydes and amines, yields α-aminonitriles (see Scheme 4).

A few years later and building on this experience, this same group applied the same protocol for the eco-friendly synthesis of α-sulfonylimidonitriles by hydrocyanation of the corresponding sulfonylimines via a one-pot two-step procedure using potassium hexacyanoferrate (II) as a cyanide source, benzoyl chloride as a promoter and potassium carbonate as a base [25]. The mechanism of the reaction is similar to that described in Scheme 4 above, except that the preformed imine precludes the formation of water, which is replaced by ethanol as the proton source.

**Scheme 5:**
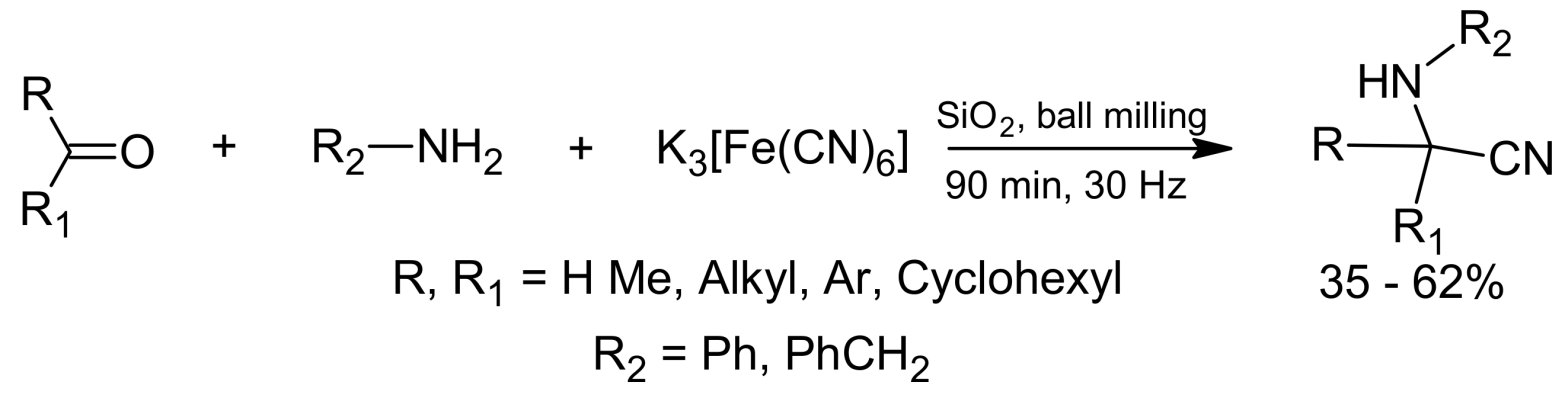
Mechanochemical Strecker synthesis of α-aminonitriles using K_3_[Fe(CN)_6_].

Cyanoferrate complexes are well known for their intrinsic stability, which sometimes can be overcome under thermal energy (>350 °C) to release the active cyanide species, in the form of HCN or cyanogen [26], under photochemical [27], chemical [22] or mechanochemical conditions. The use of mechanical energy to activate chemical reactions in the solid state, referred to as mechanochemistry, is highly appealing in efforts to search for feasible scenarios for the origin of life under a prebiotic environment. Hernández et al. [28] used this mode of activation to prepare amino acids by high-speed milling of potassium ferrocyanide or ferricyanide in the presence of silica gel and an aldehyde (or a ketone) and the relevant benzylamine (or aniline). The products were obtained in 35–62% isolated yield (Scheme 5).

Controlled experiments confirmed that gaseous HCN, generated by reaction with moisture contained in silica gel, was trapped in-situ by the Schiff base. Hydrolysis of the α-aminonitriles was achieved in a ball mill in the presence of paraformaldehyde and NaOH in 51%, thereby completing the synthesis of amino amides under conditions similar to prebiotic circumstances. From these results, the authors concluded that the mechanochemical activation of these readily available iron complexes under environmental mechanical stress could have been key for the formation of prebiotic building blocks such as α-aminonitriles or nucleobases.

This procedure to generate targets under mechanochemical activation of iron cyano complexes exhibited few limitations such as moderate yields. The method also appears restricted only to aromatic amines and no alkyl amine was used in the reaction.

In order to expand the scope of the reaction, the same group recently developed a facile protocol for Strecker reactions using a mixture of potassium ferri- and ferrocyanide in a biphasic medium [24]. The protocol consists in heating the corresponding carbonyl compound and amine to 80 °C in a biphasic solvent consisting of ethyl acetate–water (1:1) in the presence of a mixture of K_3_[Fe(CN)_6_]:K_4_[Fe(CN)_6_] (3:4). Acetic acid was added as a proton source to promote HCN release. Under these conditions, both alkyl and aryl amines including the carbonyl reaction partners could be accommodated; the products were obtained in up to 93% yield, albeit primary alkyl amines led to lower yields. This was however attributed to the retro-Strecker reaction. The biphasic solvent system avoids solubility problems, which may arise with heterogeneous conditions.

**Scheme 6:**
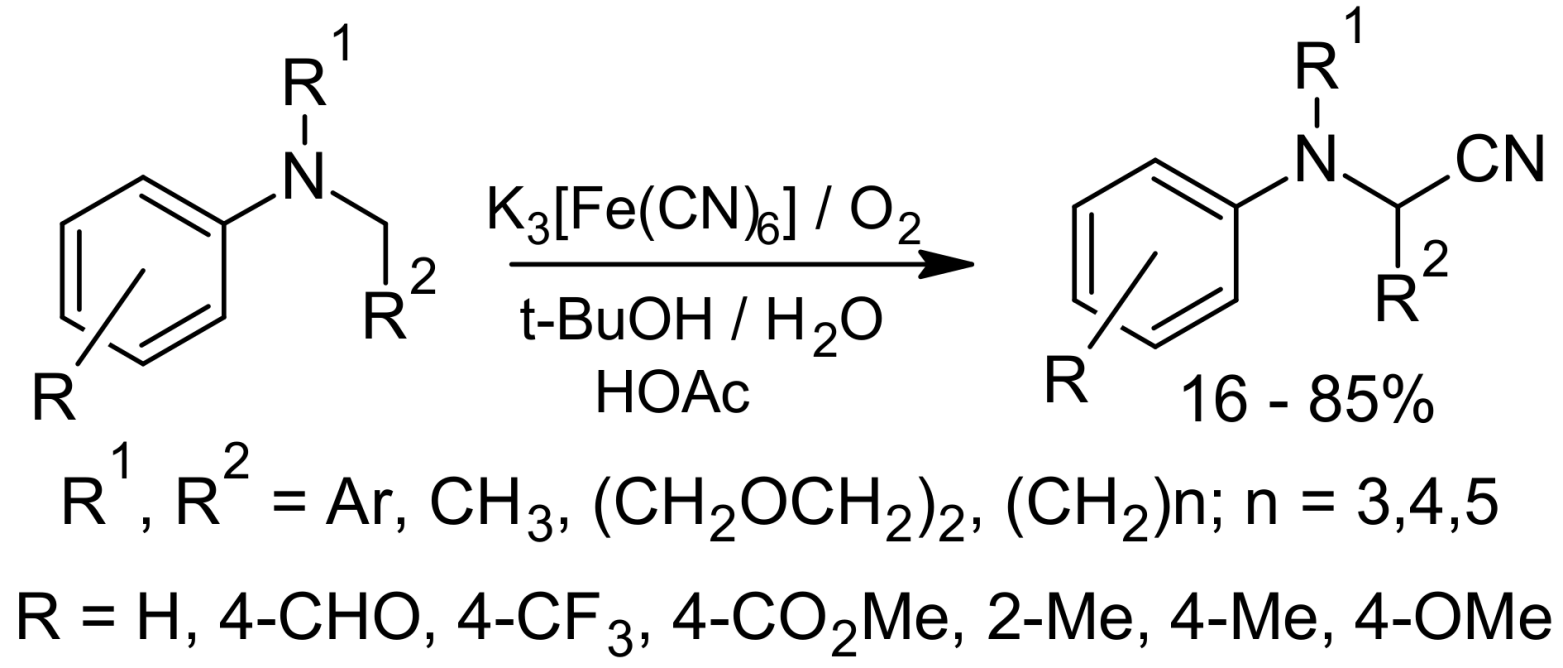
Oxidative cyanation of a tertiary amine.

Opatz et al. [29] exploited a new approach of carbon–carbon bond formation called cross-dehydrogenative coupling (CDC) to carry out the first oxidative α-cyanation of tertiary amines to afford the corresponding α-aminonitriles in a formal Strecker synthesis. The method consists in generating an iminium ion from a tertiary amine and opposing it to an in-situ generated nucleophile [30]. The reaction was carried out at 100 °C in aqueous tert-butanol in the presence of a mixture of potassium ferri- and ferrocyanide. An oxygen atmosphere was maintained throughout the reaction to ensure a recyclable catalytic system that converts the tertiary amine into the corresponding iminium ion; the later then reacts with the in-situ generated HCN to afford the product in good yields (Scheme 6).

**Scheme 7:**
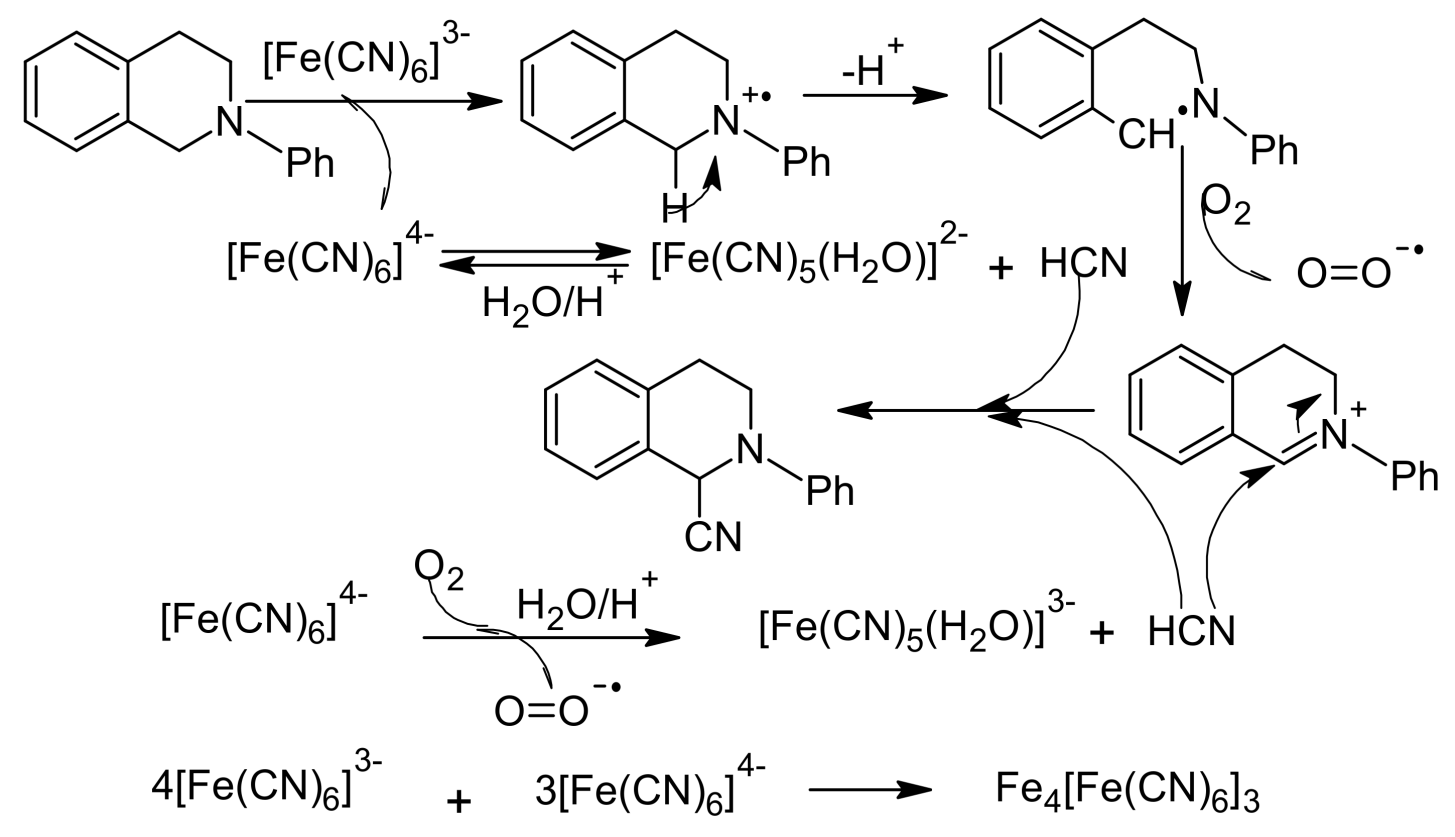
Mechanism for the α-cyanation of tertiary amines with K_3_[Fe(CN)_6_]/O_2_. −•= radical anion, +• = radical cation.

The proposed mechanism of the reaction starts with the oxidation of the amine by ferricyanide to form the corresponding iminium ion via the radical cation; the iminium ion then traps HCN generated from the ferricyanide to give the product. The presence of both cyano complexes in the correct ratio (4:3) ensures the formation of Prussian Blue, which traps any residual free cyanide, while releasing the reactive cyanide species (Scheme 7).

The scope of this operationally simple method is broad and generates Prussian Blue as the sole non-toxic co-product. Its only limitation is that it is restricted to tertiary amines.

## 4. Asymmetric Strecker Reaction

With the exception of glycine, the smallest amino acid, all other natural α-amino acids that constitute the proteins of living creatures are chiral and are found in the l-form. The classical Strecker reaction leads only to racemic mixtures, and therefore appropriate conditions must be designed to obtain homochiral products. This is done through many ways including synthesis of amino acids by fermentation, enzymatic synthesis, resolution of racemates, asymmetric syntheses [31].

Large scale industrial production of only a few amino acids is currently carried out from protein-hydrolysates, which are then extracted with a suitable solvent under carefully controlled chemical affinity pH conditions to afford products such as l-cysteine, l-leucine and l-tyrosine. Various bacteria can be used for amino acid production via fermentation, with *Corynebacterium glutamicum* and *Escherichia coli* being the most common [8,32]. The main advantage of microbial processes is the ready availability of the renewable raw materials, which are generally industrial wastes, but their bottleneck is that they highly depend on the availability of natural protein rich resources. Therefore, chemical synthesis by Strecker synthesis, though not enantioselective, remains an important method for the preparation of these biomolecules. When required, asymmetric induction as well as resolution methods allow enentioenriched products to be obtained. 

**Scheme 8:**
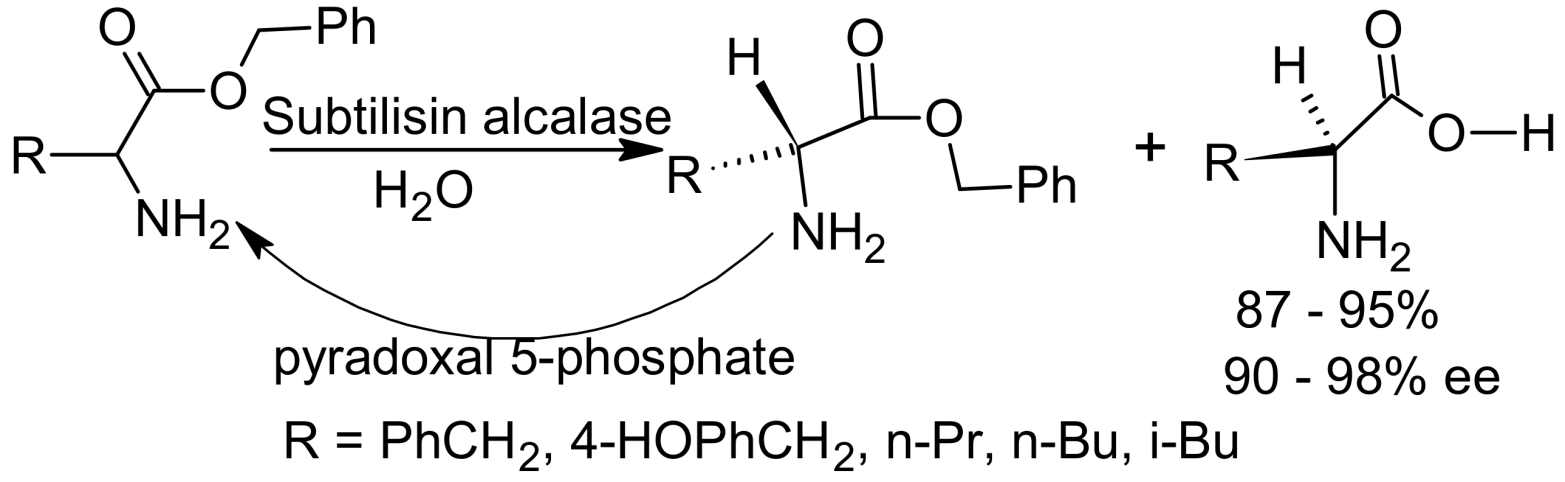
Resolution of chiral amino acids by subtilisin.

The ease of preparing racemic amino nitriles by the Strecker synthesis entails that chemical synthesis followed by enzymatic resolution remains one of the preferred methods, especially if both R and S enantiomers are needed, otherwise the unwanted isomer must be recycled via racemization to enable high yields [33]. Hydrolytic enzymes (such as α-chymotrypsin and subtilisin) and lipases are the most widely used for this purpose because of their substrate promiscuity [34]. In order to maximize the yield of the l-enantiomer, Chen et al. [35] developed a procedure for the complete conversion of a racemic amino acid into the l-enantiomer by the alcalase-catalysed resolution of the amino acid ester. The reaction takes place in a mixture of t-butanol/water and is coupled with in situ racemization of the unreacted d-enantiomer mediated by pyridoxal 5-phosphate. The l-amino acid could then be isolated in 87–95% yield and 90–98% enantiomeric excess (ee) (Scheme 8).

**Scheme 9:**
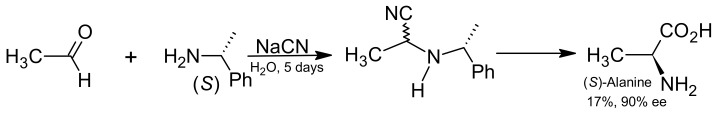
Asymmetric Strecker synthesis of (*S*)-alanine from a chiral amine.

The second most commonly used methodology for the preparation of optically active amino nitriles is through the catalytic enantioselective cyanation of chiral non racemic imines to form the corresponding diastereoisomeric amino nitriles [36], which after separation, can be further processed into the homochiral amino acids. The first example of this approach was reported in 1963 by Harada et al. [37,38] who prepared (*S*)-alanine by the reaction of acetaldehyde and (*S*)-α-phenylethylamine in the presence of an aqueous solution of sodium cyanide to give the corresponding α-amino nitrile in a diastereoselective ratio of 3.3:1; the correct diastereoisomer was finally converted into an optically enriched product in 17% overall yield and 90% ee (Scheme 9).

Carbohydrates belong to the chiral pool and are often utilised as a cheap source of chiral auxiliaries in many asymmetric syntheses. Kunz et al. [39] used 2,3,4,6-tetra-*O*-pivaloyl-β-d-galactopyranosyl amine-derived Schiff bases as chiral auxiliaries in the Strecker synthesis of the corresponding amino nitriles. The reaction selectivity could be controlled by the solvent, leading to the (*R*)-diastereoisomer (75–90%) in isopropanol or tetrahydrofuran, and to the opposite (*S*)-isomer in chloroform. 

The use of a stochiometric amount of chiral auxiliaries such as phenylethylamine and phenyl glycinol [40,41], which must be easily removed groups after the reaction, represents a limitation of the method, more so if the final product contains functional groups (such as unsaturation) that are sensitive to hydrogenation. 

**Scheme 10:**
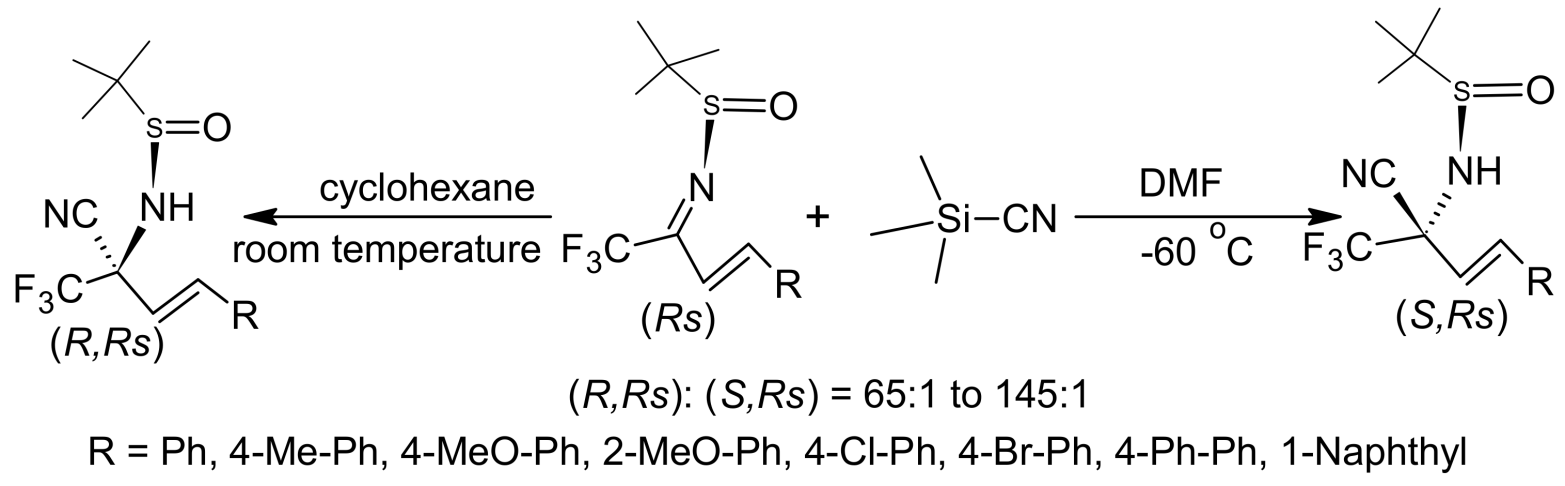
Chiral induction by tert-butylsulfinyl ketimines.

Over the past years, tert-butyl sulfinamide has emerged as an ever increasingly popular ammonia surrogate in the Strecker reaction. This popularity originates from the fact that either enantiomer of this amine is inexpensive to prepare on a large scale in enantiomerically pure form [42]. Its Schiff bases with various carbonyl compounds undergo enantiofacial cyanide addition in high diastereoisomeric ratio [43]. After separation of the two diastereoisomers, the *N*-tert-butanesulfinyl group can be conveniently cleaved by simple methanolic HCl treatment to release the corresponding amino acid hydrochloride in high ee (Scheme 10) [44,45].

**Scheme 11:**
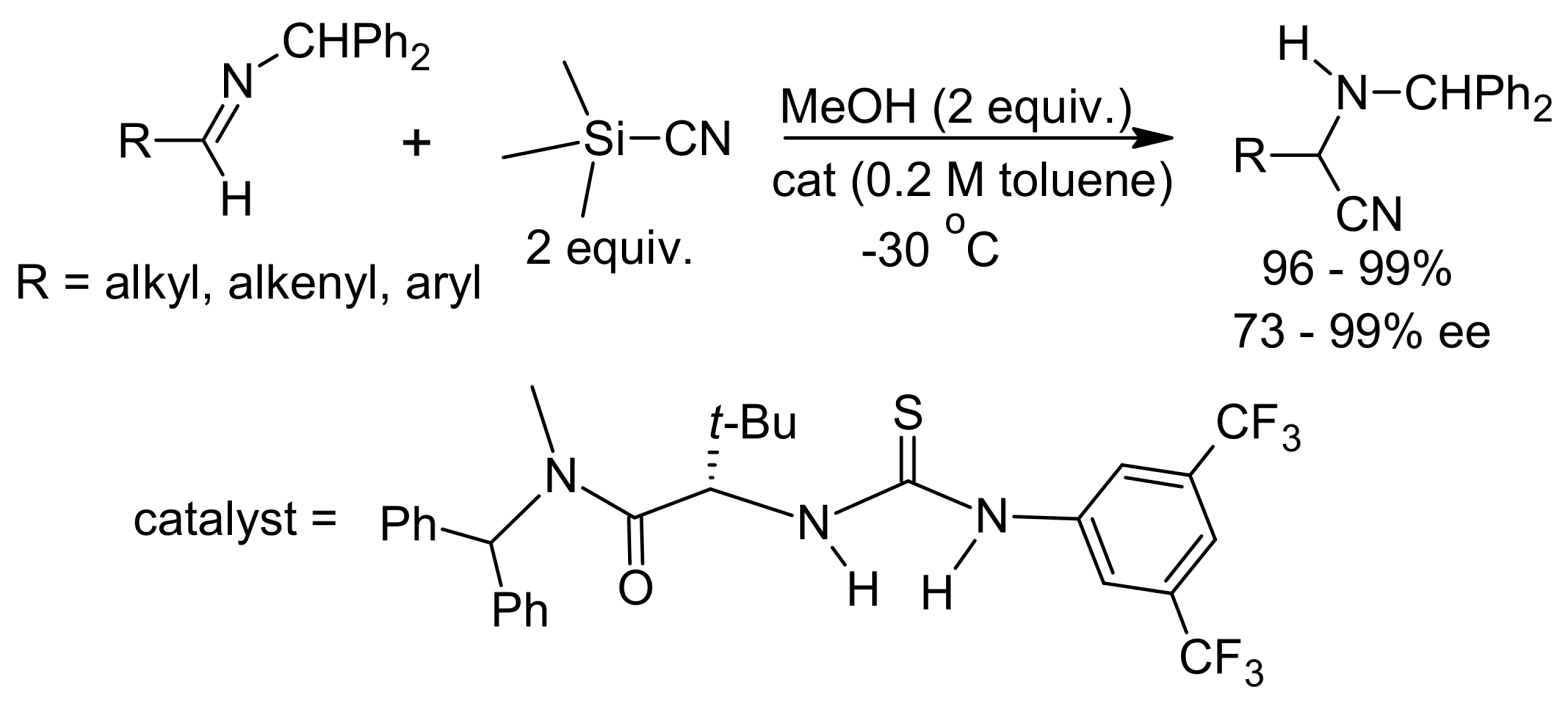
Asymmetric hydrocyanation of imines.

However, the organo-catalysed asymmetric Strecker reaction is the most straightforward and viable method for the synthesis of homochiral amino acids. Since it was first reported in 1996 [46] when a cyclic dipeptide was used as a chiral catalyst, the asymmetric Strecker synthesis has witnessed extensive development. The judicious choice of chiral auxiliary is key to the success of this strategy. One particular catalyst described by Jacobsen, allowed the syntheses of enantiomerically enriched non-proteinogenic amino acids in high isolated yields (96–99%) and high ee (73–99%) [47]. The catalyst, which directs the chiral discrimination of the hydrocyanation step, contains only one stereogenic center, is made up of a simple chiral amido-thiourea and lacks sensitive functional groups; it is used in substoechiometric amounts and is compatible with various substrates as well with the reaction conditions that involve extensive handling of cyanide for adaption to large-scale synthesis (Scheme 11).

In addition to large scale synthesis (up to 14-g scale), this methodology can be applied to the efficient syntheses of amino acids that are not readily prepared by enzymatic methods or by chemical hydrogenation.

**Scheme 12:**
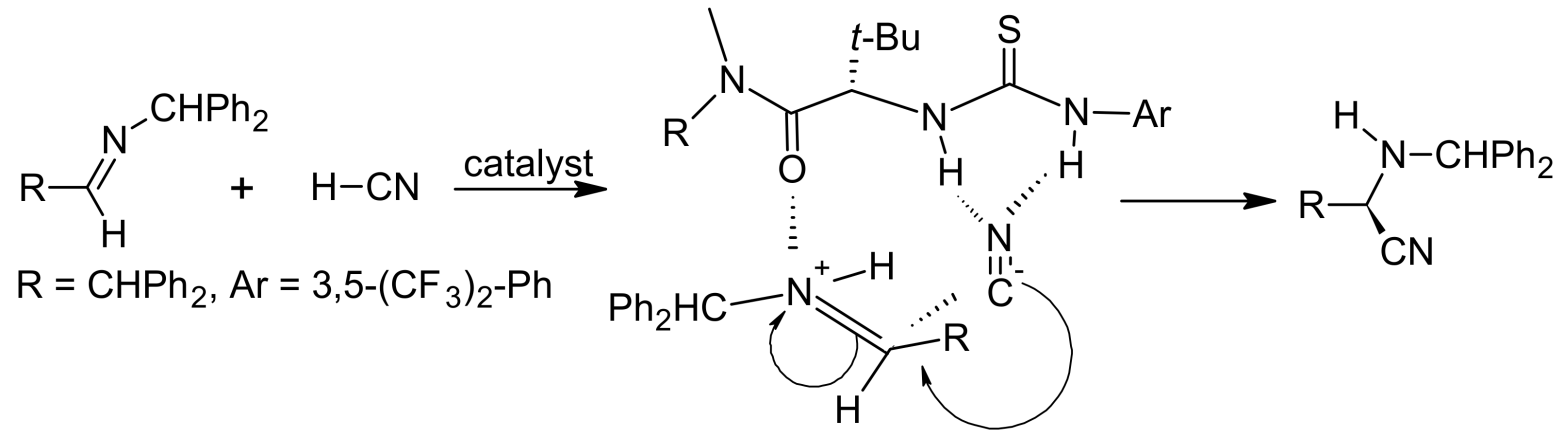
Proposed mechanism for the asymmetric hydrocyanation of imines.

The proposed mechanism of this asymmetric hydrocyanation, based on experimental results and computational data, is depicted in Scheme 12: the initially formed Schiff base is protonated by HCN, generating an iminium ion, which together with cyanide is bound to the catalyst. The collapse of this ion pair and carbon–carbon bond formation completes the formation of the product.

Prior to these catalysts, Jacobsen and his group used a chiral (salen)Al(III) complex to catalyze an array of asymmetric nucleophile addition reactions including hydrocyanation of aldimine to afford amino nitriles in good yields (69–99%) and enantioselectivity (37–99% ee) [48]. However, the (salen)Al(III) complex contains two asymmetric centers, contrary to the previous catalyst, which contains only one chiral center and is easy to prepare. 

**Scheme 13:**
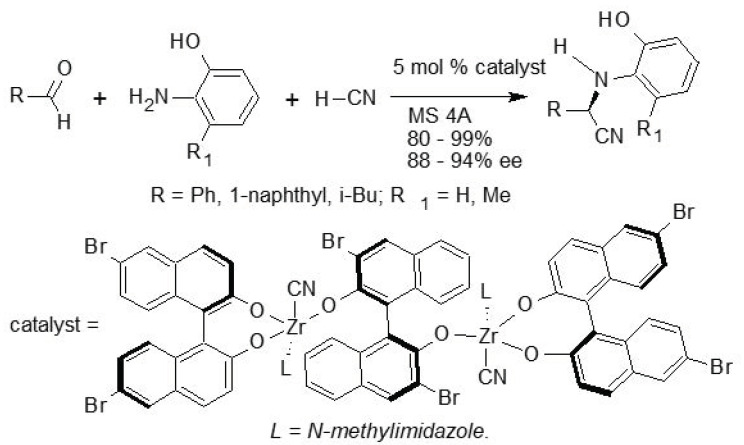
Asymmetric Strecker synthesis mediated by Kobayashi’s catalyst.

The above reactions and related procedures imply that the Schiff bases are generated in a separate step before the cyanide addition. From a multicomponent reaction standpoint, a direct mixing of all three components in the presence of the chiral catalyst would be more practical. Kobayashi and co-workers [38,49] developed a chiral Zr-complex-based catalyst that enabled the direct one-pot asymmetric synthesis of amino nitriles in high yields and ees (Scheme 13).

The main bottleneck of this approach is its limitation to the anilines as the amine components.

Many other catalysts, such as a hydroquinidine-based catalyst, have successfully mediated the Strecker reaction between benzaldehydes, morpholine and Me_3_SiCN in the presence of NaF in dichloromethane to form chiral α-amino nitriles in excellent yields (90–95%) and high enantioselectivities (80–94% ee) [38,50].

**Scheme 14:**
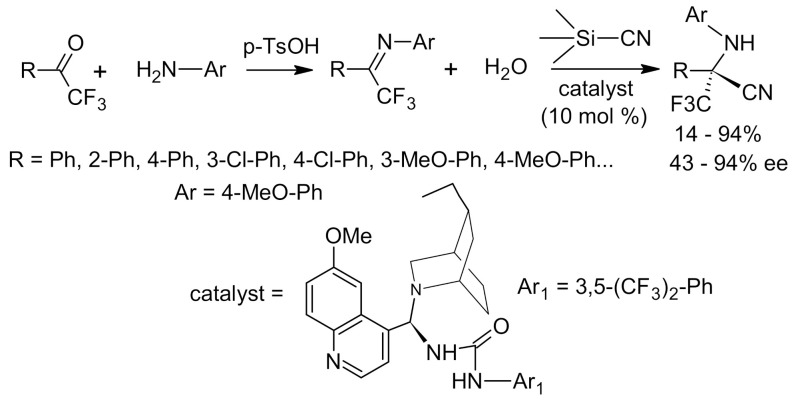
Two-step one-pot asymmetric Strecker reaction.

Fluorine containing α-amino acids bearing a quaternary α-carbon have found wide application as potential enzyme inhibitors and antitumor (antibacterial) agents [51]; unfortunately, they are difficult to prepare, especially in enantiomeric pure form because ketimine formation is more difficult than that of aldimines. One contribution to solving this challenge came from Liu et al. [52] who designed a highly enantioselective two-step one-pot facile synthesis of fluorinated Cα-tetrasubstituted amino nitriles from α-fluoroalkyl α-aryl ketones, anilines, and Me_3_SiCN. This procedure starts with the p-TsOH catalysed ketimine formation, followed by the asymmetric Strecker reaction mediated by a chiral bifunctional tertiary amine. Water generated as by-product in the first step acts a promoter and an additive in the upstream step and helps the release of HCN from Me_3_SiCN. Improved yield and enantioselectivity were achieved by this first example of tandem reactions (Scheme 14).

**Scheme 15:**
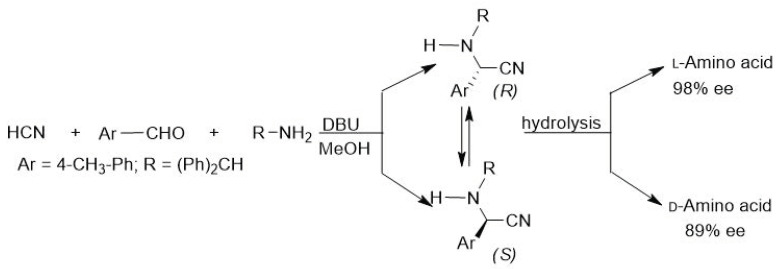
Spontaneous resolution of racemic α-amino nitriles.

The spontaneous generation of enantioenriched amino acids from achiral starting materials has been cited in numerous studies as a possible source of these L-biomolecules. In one such study by Kawasaki et al. [53], an enantioenriched α-amino nitrile was obtained by the first spontaneous crystallization with up to 96% enantiomeric excess (ee) from the reaction of the corresponding three achiral precursors: hydrogen cyanide (HCN), p-tolualdehyde, and benzhydrylamine. The Strecker reaction leads to a racemic product in solution. Its deracemisation occurs during crystallization in the presence of DBU (1,8-diazabicyclo [5.4.0]undec-7-ene) by a DBU induced retro-Strecker, which establishes an equilibrium between the forward and backward reaction. Continuous crystallisation of the enantiomer leads to the amplification of the resolution. Subsequent hydrolysis afforded an efficient way to access highly enantioenriched α-amino acids without the intervention of chiral materials (Scheme 15). Key to the process success is the formation of a conglomerate and the same reagents and reaction conditions gave the opposite enantiomorph in up to 89%ee. The authors also observed that amplification of solid-phase enantiomeric excess of enantiomers from extremely low ee (ca. 0.05% ee) to near enantiopurity (>99.5% ee) could be promoted by chiral α-amino acids.

The above procedure has the potential to become one of the most efficient ways to have access to highly enantio-enriched α-amino acids without the intervention of chiral starting materials.

As noted in the previous example, the initial imbalance in the distribution of stereoisomers may lead to process intensification and amplification with overwhelming enantioenrichment. Kawasaki and his group proposed that such an enantiomeric imbalance may arise from the molecular orientation of an achiral imine at the single-crystal face. The enantioselective addition of HCN to the enantiotopic surface of the prochiral imine from p-tolualdehyde and benzhydrylamine (see Scheme 15) led to the formation of enantioenriched α-aminonitriles, whose absolute configurations correspond to the prochirality of the starting Schiff base. Addition of HCN to the imine Re face (100) produced the l-enantiomer while addition to the Si face (−100) generated the d-enantiomer [54].

**Scheme 16:**
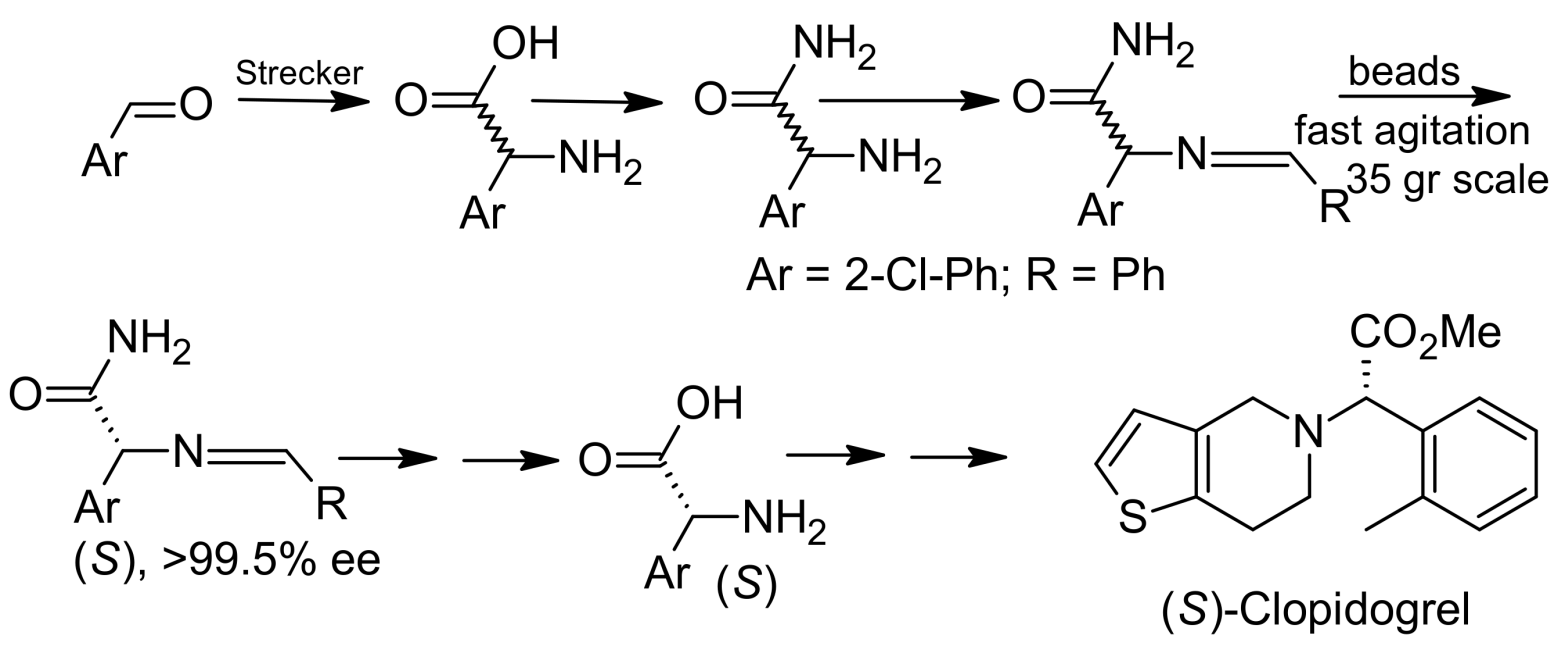
Resolution of racemic 2-chlorophenyl glycine followed by (*S*)-clopidogrel synthesis.

A racemic mixture from a Strecker reaction can be resolved into either enantiomer of chiral purity by a new concept introduced by Viedma et al. [55,56,57]. The method, called Viedma ripening, is based on chiral discrimination in the solid state simply by continuously grinding a suspension. When combined with seeding, the component that has a slight advantage starts to dominate, and the other enantiomer extinguishes gradually by racemisation in solution and attrition. Ultimately, complete optical purity is achieved. This technique was used to successfully resolve 2-chlorophenyl glycine [58], the key precursor in the synthesis of clopidogrel (Plavix), an antiplatelet medication used, among other reasons, to reduce the risk of heart disease and stroke. The racemic amino acid was first transformed to its corresponding benzylidene amide derivative to generate a conglomerate followed by Viedma ripening to afford, after seeding, the expected (S)-enantiomer in high enantiomeric excess (Scheme 16).

Many other examples of resolution of racemate using Viedma ripening and seeding can be found in references 50 and 52.

The picture of asymmetric Strecker cannot be complete without mention of enzyme-mediated homochiral amino acid synthesis. Enzymes can easily be adapted to green chemistry conditions and their inherent stereoselectivity can be exploited in the synthesis of stereo-defined, biologically active amino acids [59]. Despite the potential of enzymes as green mediators in the Strecker reaction, only one such example, based on kinetically controlled lipase-mediated transacylation, has appeared in the literature [60], leaving this area open for more development and innovation.

## 5. Petasis Reaction

**Scheme 17:**
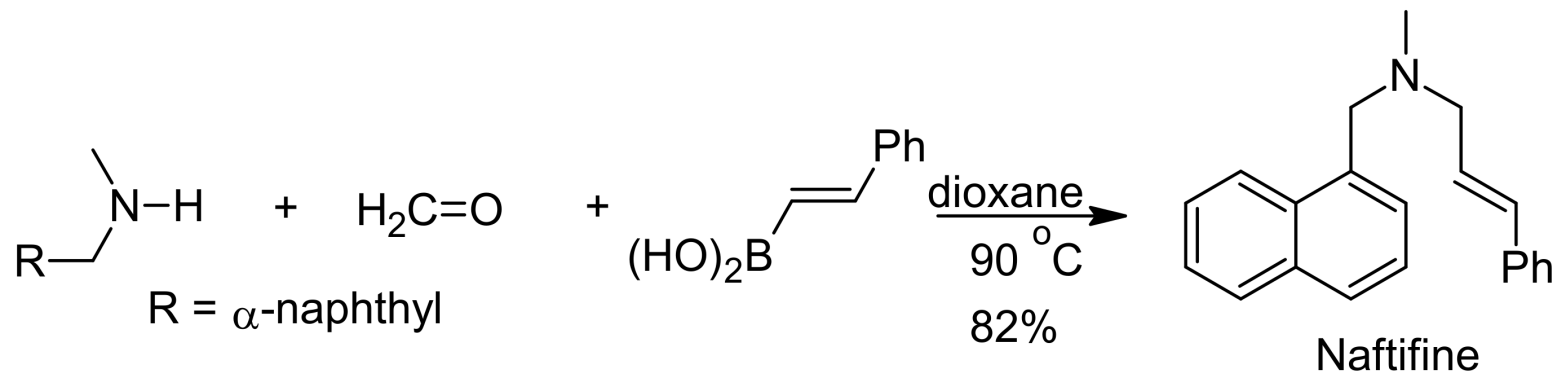
Synthesis of allyl amine by Petasis reaction.

In its original form, the Petasis reaction led to the formation of Mannich bases and was therefore termed the “Petasis borono−Mannich reaction” from allyl amines and formaldehyde, using boronic acids as the nucleophile component [11] (Scheme 17).

One important feature of this reaction is that the allylamine product is obtained with complete retention of the geometry of the olefinic bond. Thus, bioactive allyl amine products with predefined geometry, including naftifine, an antimitotic agent, could be accessed in a simple and efficient process.

This original version moved quickly beyond its original scope and developed into a powerful and convenient method for the synthesis of functionalized amine derivatives, such as β-amino alcohols, allyl amines and various heterocyclic compounds [61,62]. The introduction of glyoxylic acid as the aldehyde component brought about a new α-amino acid synthesis, which rapidly gained popularity not only because of its multicomponent nature, but also because it eliminated the use of toxic cyanide [63] in the synthesis of these important compounds.

**Scheme 18:**
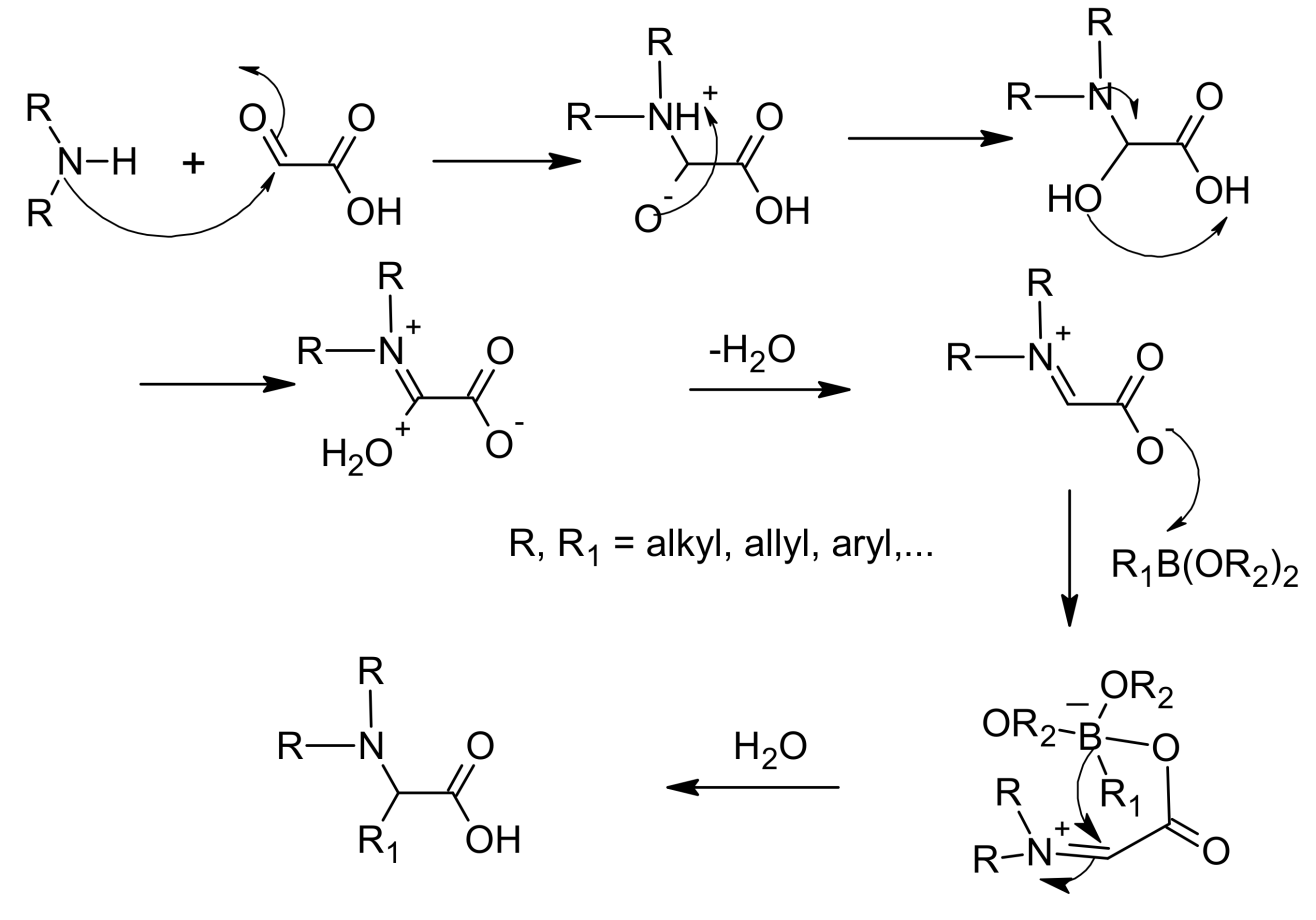
Mechanism of the Petasis amino acid synthesis.

The reaction mechanism proceeds via the formation of an imine or an iminium species, from the condensation of the amine with glyoxylic acid. Reaction of this intermediate with the boronic acid (or ester) produces a tetracoordinate boronate intermediate, followed by the intramolecular delivery of the organic group to the iminium carbon, forming the product (Scheme 18). This mechanism shows that the presence of a free adjacent hydroxyl group, which reacts with the boronic acid to form a more nucleophilic ate complex, is critical for the success of the reaction.

**Scheme 19:**
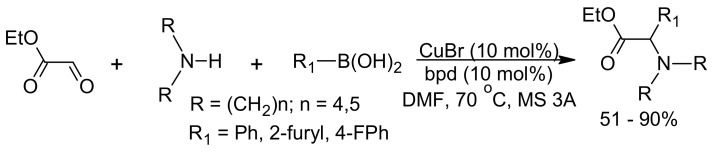
Cu(I)-catalysed Petasis synthesis of Ethyl glycinates.

Where this OH group is absent, acid catalysts [64] and organocatalytic systems may be required to speed up the reaction. Accordingly, a cuprous bromide-catalysed Petasis reaction was developed by Bergin et al. [65] for the synthesis of ethyl glycinate derivatives from the tertiary amines and boronic acids in the presence of ethyl glyoxylate (Scheme 19).

The reaction proceeded smoothly in DMF in the presence of 2,2′-bipyridine (bpd) as the ligand and molecular sieves 3 Å with the exclusion of oxygen and moisture to afford the expected amino acid esters in good to excellent yields of up to 90%.

**Scheme 20:**
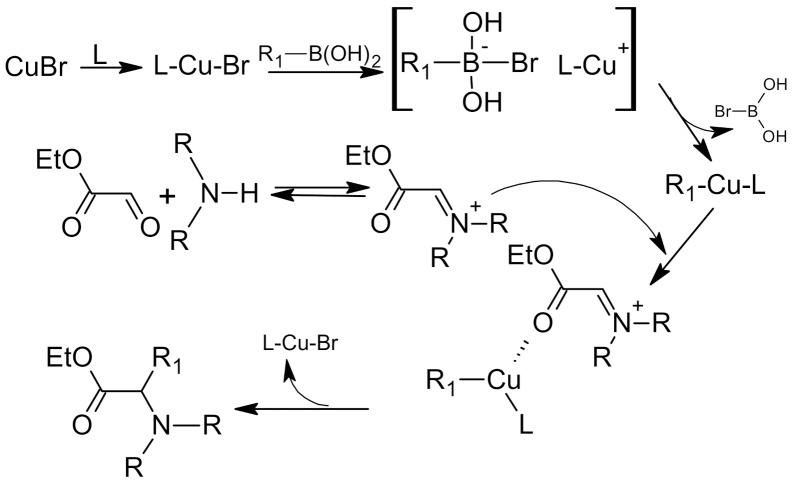
Mechanism of Cu(I)-catalysed Patasis reaction of ethyl glyoxylate with amines and boronic acids.

The mechanism postulated starts with the initial formation of a boronate salt through the interaction of the boronic acid. This complex then transmetallates copper, leading to the formation of an organocuprate, followed by carbon–carbon bond formation through nucleophilic attack at any iminium ions in solution (Scheme 20). All the postulated boronic species including the ate complex were identified by B-NMR, confirming the mechanism involved in the reaction.

The Petasis reaction is flexible and appropriate for solid phase synthesis and was applied in efficient protocols for the rapid generation of libraries of biologically promising β-lactams [66] including DNA-encoded libraries and DNA-tagged α-aryl glycines [67].

**Scheme 21:**
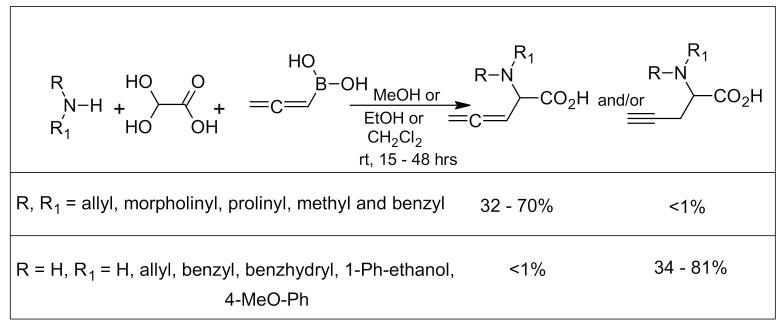
Synthesis of α-propargyl and/or α-allenyl α-amino acids.

Apart from vinyl boronic acids, allenyl, aryl boronic acids and other heterocyclic derivatives can also be used in Petasis multicomponent coupling. Possible substrate scope includes thienyl, pyridyl, furyl, benzofuranyl, 1-naphthyl, and aryl groups with either electron-donating or electron-withdrawing substituent [68]. In the case of allenyl boronics acid or allenyl pinacolboronates, their one-step, three-component condensation with amines and aldehydes affords, depending on the reaction conditions, α-allenyl or α-propargyl α-amino acids [69], indicative of a possible competition between the original allenyl substituent and the corresponding propargyl group, its rearrangement analogue, without acid or metal catalysis. The authors noted that most primary amines including anilines exclusively formed the α-propargyl products, while more bulky primary amines were less selective. Secondary amines exclusively gave the α-allenyl products (Scheme 21).

**Scheme 22:**
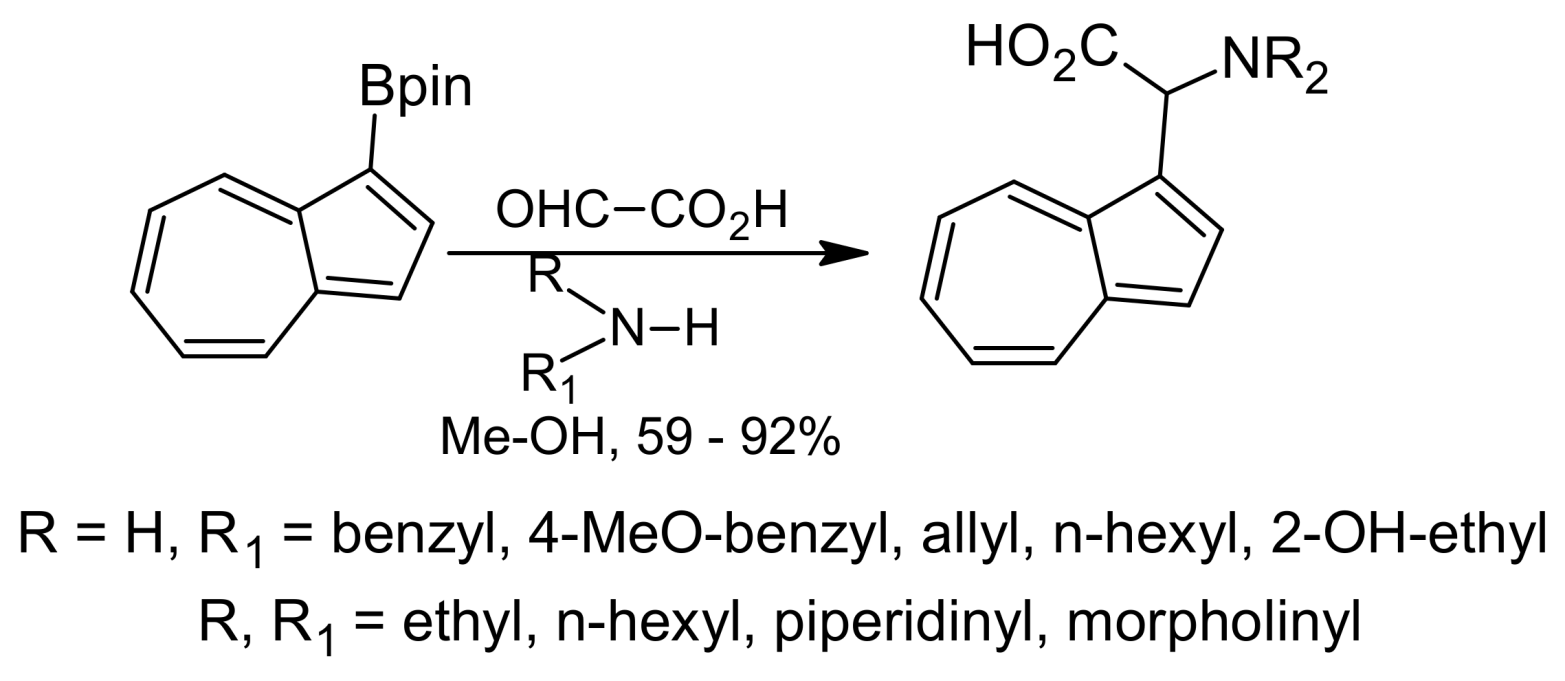
Synthesis of azulenylglycine derivatives by Petasis reaction.

The versatility of the Petasis reaction can be further illustrated by the synthesis of azulenylglycine derivatives from the corresponding azulen-2-ylboronic acid pinacol ester [70] (Scheme 22). Indeed, because the azulene structure can impart its colour to a host molecule, azulenyl peptides are an attractive target as a diagnostic tool to study conformational changes in peptomimetics and peptide chemistry. Such specific molecular structures are very limited, and this protocol constitutes one of the few methods that allow the introduction of the azulen-1-yl group onto the α-carbon of N-alkylglycines under mild conditions.

Apart from glyoxylic acid, pyruvic acid and α-ketoacids can be used as the carbonyl component [68,71]. The reaction is compatible with a wide range of amine components, such as primary and secondary alkyl amines, anilines, amino acids, peptides, hydrazines and hydroxylamines, sulfonamides and sulfinamides, and glucosamine derivatives [72], leading to the synthesis of the corresponding glycine derivatives, including a few examples in which free ammonia has been shown to successfully participate in the reaction [69,73,74,75,76].

**Scheme 23:**
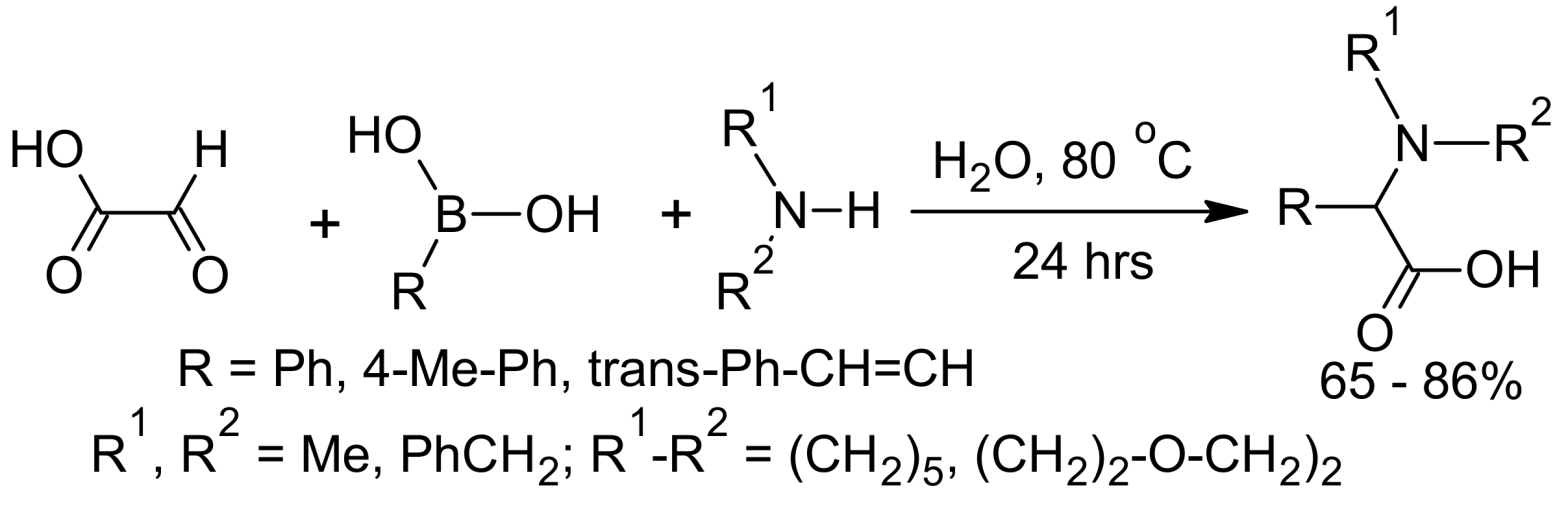
Petasis amino acid synthesis in water.

Because boronic acids are generally stable and soluble in aqueous media, they can be considered ideal partners in the Petasis reaction in water as solvent since the multiple hydrophobic reactants are brought in closer proximity due to hydrophobic interactions. Water is considered as the green solvent par excellence because it is cheap and readily available; it is non-toxic, non-inflammable, and environmentally friendly. Based on these considerations, Gois and his group developed a Petasis amino acid synthesis in water as the solvent (Scheme 23). The reaction proceeded well with both primary and secondary amines including sterically hindered amines such as adamantylamine [77].

The negative transition molar volume as well as the hydrophobic effect brought about by water as a solvent appear to be the driving force behind the success of this protocol.

**Scheme 24:**
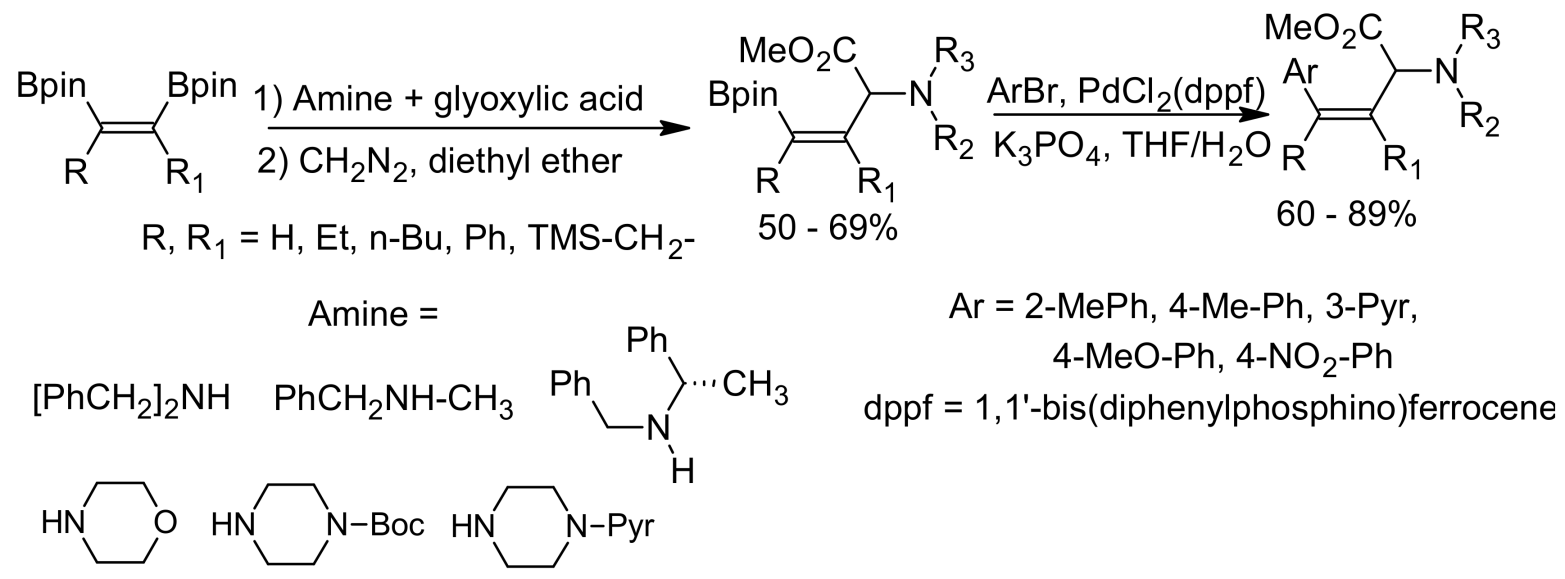
Synthesis of γ-unsaturated amino esters by Petasis reaction followed by Suzuki coupling.

Vicinal Z-alkene diboronic esters, readily accessible by reaction of the corresponding alkynes with bis(pinacolato)diboron (pin) in the presence of an appropriate catalyst [78], offer the opportunity of a double Petasis reaction on these alkenes. Though no products resulting from a double Petasis condensation were detected under the experimental conditions, the corresponding γ-boronated unsaturated amino acids were obtained in good yields. These resulting amino esters were then subjected to Suzuki coupling with the second boronate moiety to give diversely substituted olefinic amino acid systems [79] (Scheme 24). In most cases, this tandem protocol led to the formation of the final product with retention of alkene configuration to afford the Z isomers as single isomers.

## 6. Asymmetric Petasis Reaction

**Scheme 25:**
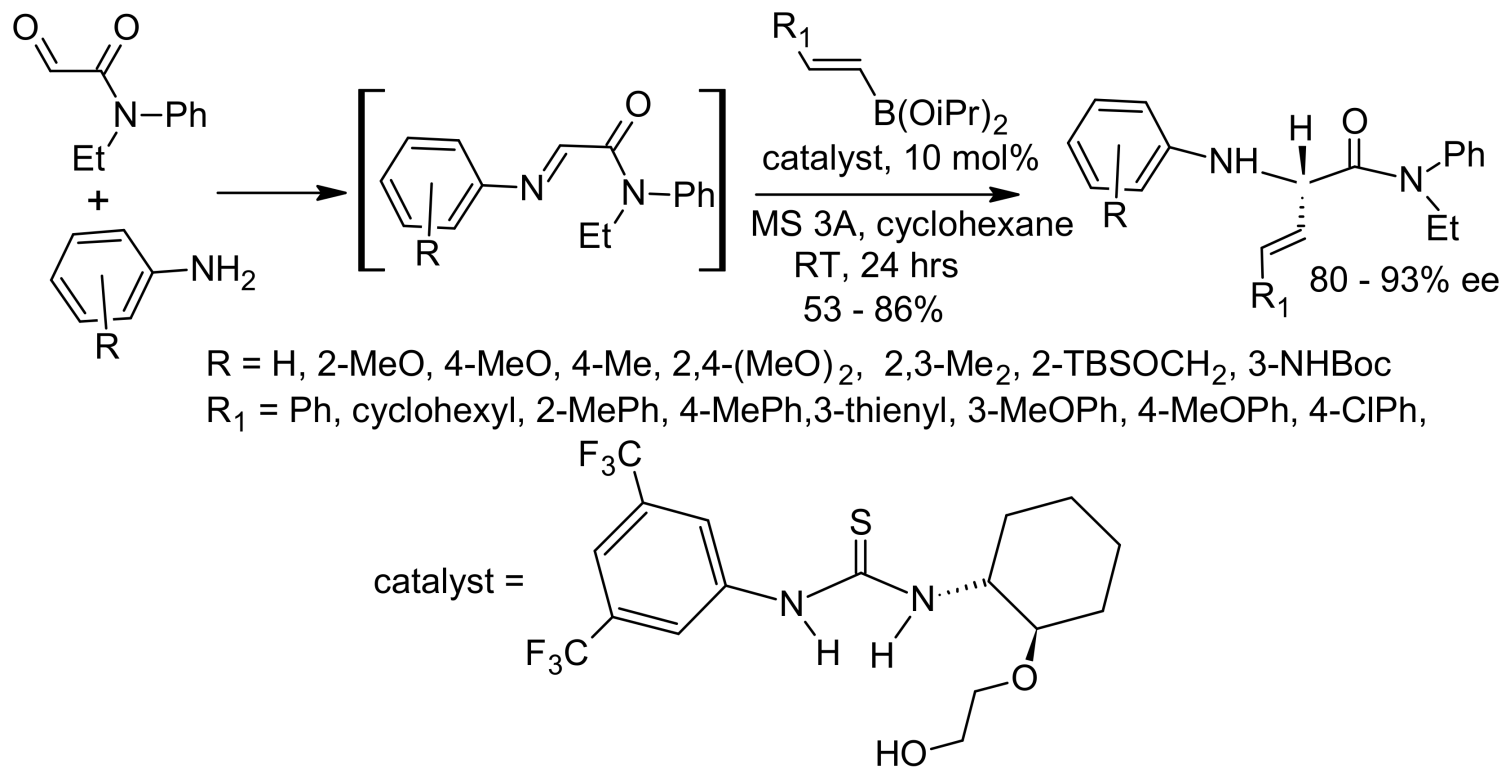
Catalytic asymmetric amino acid synthesis.

The asymmetric version of the Petasis reaction is relatively recent, dating back only to 2007 when a new designed thiourea-based catalyst [80] (Scheme 25), followed by biphenol-based [81] catalysts, were employed for the first time in the asymmetric induction in this reaction. High yields (71–87%) and ees (93–97%) were achieved. The asymmetric Petasis reactions employing *N*-benzylphenylglycinol as the chiral amine were reported to proceed with poor diastereoselectivity [82]. Chiral tert-butylsulfinamide has been used as the amine component in many Strecker reactions to produce homochiral amino acids (Scheme 10). Accordingly, this amide was also utilised as the amine partner in the Petasis reaction, providing rapid access to β,γ-unsaturated amino acid derivatives in high yield and diastereoselectivity [83]. The main advantage of this method is the mild deprotection conditions needed to release the free amino acid. Various Lewis acids may occasionally be used to speed up the reaction rate [84].

Inokuma et al. [85] developed a thiourea-based catalyst, which efficiently effected the asymmetric induction of the Petasis reaction of *N*-aryl-α-imino amides and vinyl boronates in 53–86% yield and 80–93% ee (Scheme 25). This process can be used not only for the asymmetric synthesis of unnatural amino acid derivatives but also for the stereoselective synthesis of modified dipeptides and tripeptides.

**Scheme 26:**
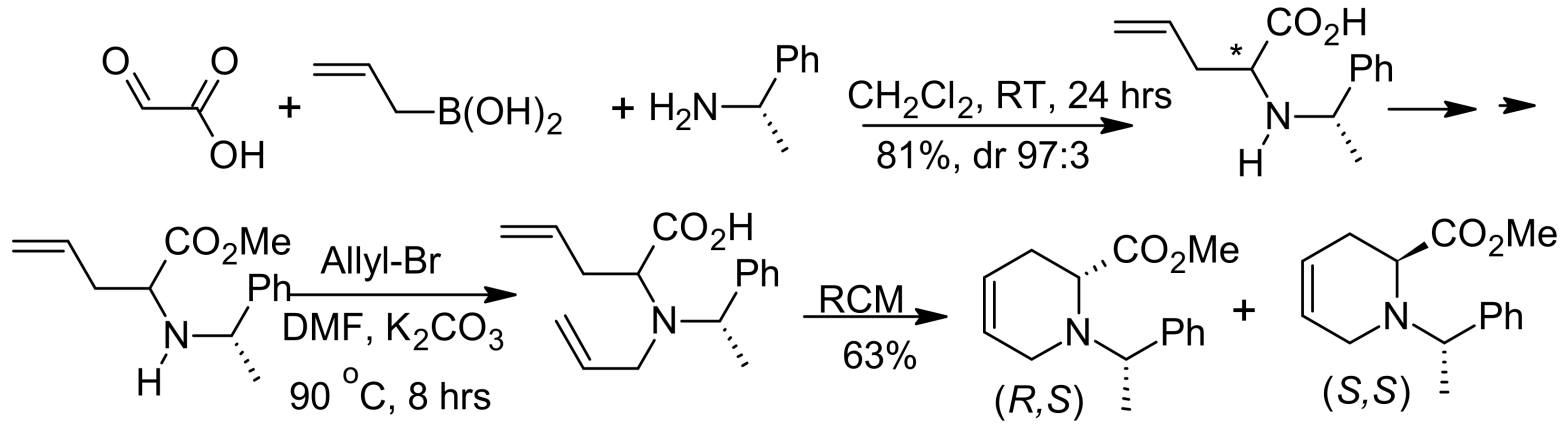
Petasis reaction followed by ring-closure metathesis. * signifies an asymmetric center.

The preservation of the unsaturation integrity of the boronate is one of the key requirements in certain protocols because the product double bond can participate in another process such as ring closure metathesis (RCM) to form cyclic amino acids. This is the novel approach used by Morozova et al. [86] to synthesize enantiopure (*R*)- and (*S*)-pipecolinic acids from allylboronic acid using (*S*)-α-methylbenzylamine as a chiral auxiliary in the Petasis reaction. The corresponding allylglycine derivatives were obtained in good yield and high diastereoselectivity. Subsequent esterification, *N*-allylation followed by ring-closing metathesis enabled the preparation of the enantiomerically pure cyclic α-amino acid derivatives (Scheme 26).

The main disadvantage of the method is the necessary hydrogenolytic conditions for removal of the *N*-benzylic group, which also reduces the olefin functionalities, which might be useful in other transformations, leading to the corresponding saturated derivatives.

**Scheme 27:**
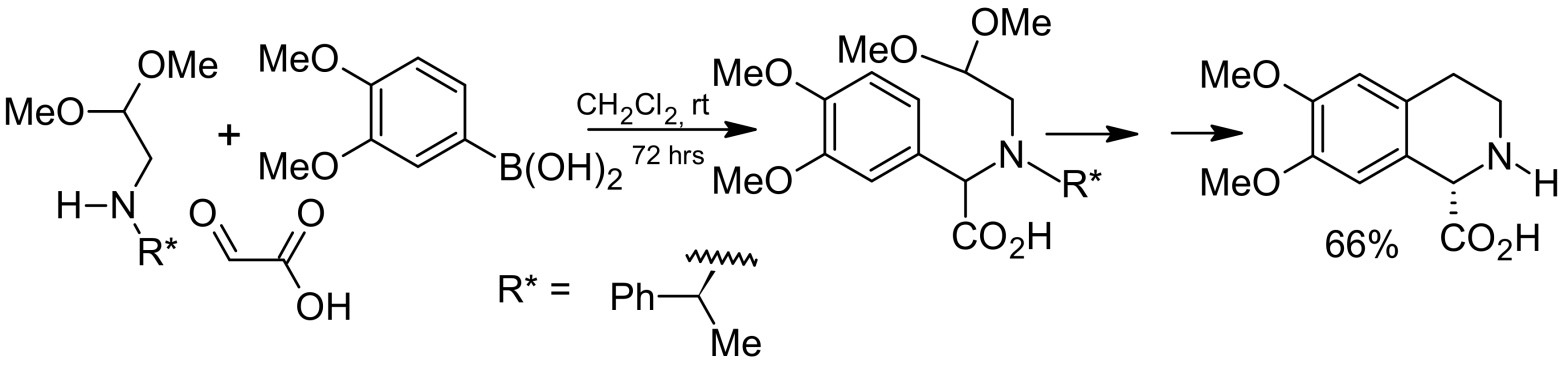
Synthesis of (+)-6,7-dimethoxy-1,2,3,4-tetrahydroisoquinoline-1-carboxylic acid. * indicates that this substituent is chiral and its stereochemistry is specified in the definition of R*.

An additional functionality in the amine partner can be utilised to allow further chemical manipulation of the products to form more complex structures, such as (+)-6,7-dimethoxy1,2,3,4-tetrahydroisoquinoline-1-carboxylic acid, whose synthesis was accomplished by the Petasis reaction from amino acetaldehyde dimethyl acetal followed by the Pomeranz–Fritsch–Bobbitt cyclization to afford the corresponding tetrahydroisoquinoline derivatives. The final product was obtained in 66% overall yield and 90% ee [87] (Scheme 27).

In principle, enantiomerically pure boronic esters, which are readily accessible from the straightforward condensation of the corresponding vinylboronic acid with a set of commercially available chiral 1,2-diols in high yields and purity, can be considered an attractive alternative as chiral partners with achiral amines and carbonyl compounds. Unfortunately, earlier studies resulted in poor selectivities (~15% ee) [88] and further investigations using more chiral vicinal diols will be needed to improve the selectivity.

## 7. Discussion

As multi-component reactions, both Petasis and Strecker amino acid syntheses lead not only to the preparation of amino acids as the end products in a single operation, but also to the formation of two new bonds: a carbon–nitrogen bond and a carbon–carbon bond, an important operation towards the construction of densely substituted structures.

Due to the simplicity in its practical operation, which requires only a simple mixing of the easily available acetaldehyde, ammonia, and hydrogen cyanide for a fixed period, the Strecker reaction is indisputably the method of choice for the direct synthesis of α-amino nitriles from its three reacting components. Subsequent hydrolysis of the amino nitriles leads, in a one-pot two-step process, to the expected α-amino acids.

As most cyanide sources are cheap industrial raw materials, the widespread application of the Strecker reaction hinges on the starting materials aldehydes and ketones, the carbonyl component of the reaction, which are readily available with different substitutions, to afford free amino acids in unlimited amount from ammonia, and N-protected amino acids from substituted amines.

Thermodynamically, the conventional Strecker synthesis inherently lacks selectivity, forming both R and S enantiomers in equal amount. Access to either optical isomer is however straightforward through enzymatic or chemical resolution, as well as asymmetric syntheses.

A unique advantage of the Strecker reaction can be demonstrated by the synthesis of labelled carboxylic acid groups of amino acids with carbon-11 (see Scheme 3) and carbon-14 [20], which are more conveniently introduced by this reaction.

On the other hand, allenyl carbonyl compounds are not very common and, when available, they are not cheap. Thus, the synthesis of allenyl amino acids by Strecker reaction is virtually non-existent.

The use of toxic cyanide species remains the major setback of the reaction. Nevertheless, the introduction of harmless cyanoferrate complexes as replacement of toxic cyanides indicates that the Strecker synthesis will continue to enjoy unrestricted applications.

In contrast, the Petasis reaction is a one-pot one-step process leading directly (without the hydrolysis step) to the expected final products. Both boronic acids and esters are easily accommodated without significant adverse effect on the reaction yield. Where boronic acids are the starting materials, the by-product boronic acid (or its derivative) is generally water soluble and non-toxic and can be easily removed from the product or recovered for reuse, making the whole process atom economic and environmentally benign. In addition, the absence of any cyanide component in the reaction is a great advantage over the classical Strecker synthesis.

Compared to the Strecker reaction, the Petasis reaction has other distinctive characteristics. First, geometrically pure vinyl boronic acids are easily prepared by hydroboration of alkynes with various alcohols, such as methanol, isopropanol, pinacol, and catechol, and can lead to unlimited synthesis of amino acids. Furthermore, the amino acid products are obtained, after the reaction, without any erosion of the geometric integrity of the starting alkene moiety. With continuous progress being achieved in the preparation of alkylboronates from the corresponding alkenes [89,90,91] and alkyl halides [92,93], the Petasis reaction can virtually allow unlimited access to designer amino acids.

Unlike the Strecker reaction, glyoxylic acid is not toxic and equally leads to both natural and unnatural α-amino acids in a single step, while avoiding the use of toxic starting materials, which necessitate a careful elimination of the resulting impurities and by-products.

Though both reactions led to racemic products, the Petasis reaction is inherently anti diastereoselective when used with α-hydroxy aldehydes in the presence of amino acids as the amine component, leading preferentially to the formation of anti β-amino alcohols. However, appropriate catalysts are able to reverse this tendency to allow a synthetic route to the full matrix of stereoisomeric products [94].

Like the Strecker reaction, there is essentially no limitation on the amine partners, accommodating both primary and secondary amines, and laying the basis for chiral primary amines such as α-phenylethylamine and phenyl glycinol to be used as chiral auxiliaries in asymmetric syntheses. Their removal by hydrogenolysis can however be incompatible with the presence of alkene groups, which may be earmarked for further reactions. One possible constraint of the Petasis reaction seems to be the availability of α-keto acids. Surprisingly, oxaloacetic acid, a special α-keto acid that can lead to variously substituted aspartic acids, has never been used as the carbonyl component in this reaction.

As for the enantioselective synthesis of amino acids, conditions for the organo-catalysed asymmetric syntheses are similar for both reactions.

While reactive cyanide species function as the nucleophile in the Strecker reaction, boronic acids and esters act as mild nucleophilic species in the Petasis reaction and are tolerant to many reaction conditions, including those in aqueous solutions, allowing much greater flexibility in the choice of reagents and providing a wider reaction scope.

Compared to the other multitude of amino acid synthesis methods, such as the alkylation of glycine-derived Schiff bases [95], Petasis and Strecker reactions are the most direct, convergent and atom economic and occur under mild reaction conditions without transition-metal catalysts. Both reactions are valuable approaches to the same general targets from different perspectives, complementing each other, and offer two additional versatile tools in the toolbox of the synthetic organic chemist.

## 8. Conclusions

The synthesis of α-amino acids, either in their racemic or enantiomerically pure form, continues to be the focus of extensive research owing to their importance in various areas of life and physical sciences. While a multitude of protocols for their preparation have been developed, the Petasis and Strecker reactions stand out as the most straightforward and have greatly contributed to the expansion of the field, making countless amino acids available in their nature and diversity.

The two reactions are similar in many aspects: they both form the same product and are both three-component reactions using the same starting materials except the third, nucleophilic species reagent. Whereas the Strecker reaction makes use of usually toxic cyanides, except cyanoferrate complexes, which are harmless and eco-friendly, the Petasis reaction employs boronic compounds, which are generally non-toxic, easy to handle and stable to air while maintaining their reactivity towards the iminium ions.

Hydrogen cyanide, the nucleophile component in the Strecker reaction, is at the heart of all discussions regarding one of the most fundamental questions of humankind: the origin of life on earth. Indeed, HCN is mentioned as the starting material that gave rise to extant amino acids by Strecker synthesis, along with peptides and nucleic acids [96]. Under prebiotic conditions, a Strecker reaction produced racemic amino acids, whose bias towards one enantiomer and its amplification by any of the mechanisms outlined above, would lead to the overrepresentation of the L-enantiomeric form, forming the current proteinogenic amino acids.

Boronic acids, on the other hand, are abiotic [97] and are not found in nature and cannot be associated with the formation of natural amino acids through the Petasis reaction.

Nevertheless, both reactions provide us, in tandem and from readily available feedstocks, in a simple, convergent protocol, a rapid entry into these diverse important biomolecules, both natural and non-natural, racemic and enantiomerically pure, whose incorporation into peptides and other more complex molecules brings structure variability and complexity, as well as well-defined physical and biological properties.

## References

[1] Bada J., Gargaud M., Amils R., Quintanilla J.C., Cleaves H.J., Irvine W.M., Pinti D.L., Viso M. (2011). Strecker Synthesis. Encyclopedia of Astrobiology.

[2] Strecker A. (1850). Ueber die künstliche Bildung der Milchsäure und einen neuen, dem Glycocoll homologen Körper. Justus Liebigs Ann. Der Chem..

[3] Strecker A. (1854). Ueber einen neuen aus Aldehyd-Ammoniak und Blausäure entstehenden Körper. Justus Liebigs Ann. Der Chem..

[4] Pascal R., Gargaud M., Amils R., Quintanilla J.C., Cleaves H.J., Irvine W.M., Pinti D.L., Viso M. (2011). Bücherer–Bergs Synthesis. Encyclopedia of Astrobiology.

[5] Monteiro J.L., Pieber B., Corrêa A.G., Kappe C.O. (2016). Continuous synthesis of hydantoins: Intensifying the Bucherer–Bergs reaction. Synlett.

[6] Ashe K., Fernández-García C., Corpinot M.K., Coggins A.J., Bučar D.-K., Powner M.W. (2019). Selective prebiotic synthesis of phosphoroaminonitriles and aminothioamides in neutral water. Commun. Chem..

[7] Williams R.M., Hendrix J.A. (1992). Asymmetric synthesis of arylglycines. Chem. Rev..

[8] Ivanov K., Ivanova S., Georgieva M., Atanasov P. (2014). Production and regulatory analytical control of amino acids include in food additives. Pharmacia.

[9] Yasufumi O., Tetsuro S. (2003). Asymmetric Strecker Route toward the Synthesis of Biologically Active α,α-Disubstituted α-Amino Acids. Bull. Chem. Soc. Jpn..

[10] Arasappan A., Venkatraman S., Padilla A.I., Wu W., Meng T., Jin Y., Wong J., Prongay A., Girijavallabhan V., George N.F. (2007). Practical and efficient method for amino acid derivatives containing β-quaternary center: Application toward synthesis of hepatitis C virus NS3 serine protease inhibitors. Tetrahedron Lett..

[11] Petasis N.A., Akritopoulou I. (1993). The boronic acid mannich reaction: A new method for the synthesis of geometrically pure allylamines. Tetrahedron Lett..

[12] Hu X., Ma Y., Li Z. (2012). Eco-friendly synthesis of α-aminonitriles from ketones in PEG-400 medium using potassium Hexacyanoferrate(II) as cyanide source. J. Organomet. Chem..

[13] Ivon Y.M., Tymtsunik A.V., Komarov I.V., Shishkin O.V., Grygorenko O.O. (2015). Synthesis of a 2, 5-Diazabicyclo [2.2. 1] heptane-Derived α, β-Diamino Acid. Synth. Stuttg..

[14] Van Hijfte L., Heydt V., Kolb M. (1993). A versatile entry into the synthesis of α-(monofluoromethyl) amino acids: Preparation of α-(monofluoromethyl) serine and (E)-dehydro-α-(monofluoromethyl) ornithine. Tetrahedron Lett..

[15] Razafindrabe C.R., Aubry S., Bourdon B., Andriantsiferana M., Pellet-Rostaing S., Lemaire M. (2010). Synthesis of (±)-phthalascidin 650 analogue: New synthetic route to (±)-phthalascidin 622. Tetrahedron.

[16] Myers A.G., Kung D.W. (2000). One-Step Construction of the Pentacyclic Skeleton of Saframycin A from a “Trimer” of α-Amino Aldehydes. Org. Lett..

[17] Aoki K., Ijima T., Kamiyama H., Kamiko K., Terauchi Y. (2015). Anagliptin decreases serum lathosterol level in patients with type 2 diabetes: A pilot study. Expert Opin. Pharmacother..

[18] Augeri D.J., Robl J.A., Betebenner D.A., Magnin D.R., Khanna A., Robertson J.G., Wang A., Simpkins L.M., Taunk P., Huang Q. (2005). Discovery and Preclinical Profile of Saxagliptin (BMS-477118):  A Highly Potent, Long-Acting, Orally Active Dipeptidyl Peptidase IV Inhibitor for the Treatment of Type 2 Diabetes. J. Med. Chem..

[19] Xing J., Brooks A.F., Fink D., Zhang H., Piert M.R., Scott P.J., Shao X. (2017). High-yielding automated convergent synthesis of no-carrier-added [11C-carbonyl]-labeled amino acids using the Strecker Reaction. Synlett Acc. Rapid Commun. Synth. Org. Chem..

[20] Song F., Salter R., Weaner L.E. (2015). A short synthesis of d-[1-14C]-serine of high enantiomeric purity. J. Label. Compd. Radiopharm..

[21] Bandak D., Babii O., Vasiuta R., Komarov I.V., Mykhailiuk P.K. (2015). Design and synthesis of novel 19F-amino acid: A promising 19F NMR label for peptide studies. Org. Lett..

[22] Li Z., Ma Y., Xu J., Shi J., Cai H. (2010). One-pot three-component synthesis of α-aminonitriles using potassium hexacyanoferrate(II) as an eco-friendly cyanide source. Tetrahedron Lett..

[23] Poliakoff M., Licence P. (2007). Green chemistry. Nature.

[24] Grundke C., Opatz T. (2019). Strecker reactions with hexacyanoferrates as non-toxic cyanide sources. Green Chem..

[25] Li Z., Li R., Zheng H., Wen F., Li H., Yin J., Yang J. (2013). Hydrocyanation of sulfonylimines using potassium hexacyanoferrate(II) as an eco-friendly cyanide source. J. Braz. Chem. Soc..

[26] Pechenyuk S.I., Domonov D.P., Shimkin A.A., Ivanov Y.V. (2015). Thermal decomposition of iron cyano complexes in an inert atmosphere. Russ. Chem. Bull..

[27] Kuhn D.D., Young T.C. (2005). Photolytic degradation of hexacyanoferrate (II) in aqueous media: The determination of the degradation kinetics. Chemosphere.

[28] Bolm C., Mocci R., Schumacher C., Turberg M., Puccetti F., Hernández J.G. (2018). Mechanochemical Activation of Iron Cyano Complexes: A Prebiotic Impact Scenario for the Synthesis of α-Amino Acid Derivatives. Angew. Chem..

[29] Nauth A.M., Otto N., Opatz T. (2015). α-Cyanation of Aromatic Tertiary Amines using Ferricyanide as a Non-Toxic Cyanide Source. Adv. Synth. Catal..

[30] Li Z., Bohle D.S., Li C.-J. (2006). Cu-catalyzed cross-dehydrogenative coupling: A versatile strategy for C–C bond formations via the oxidative activation of sp3 C–H bonds. Proc. Natl. Acad. Sci. USA.

[31] D’Este M., Alvarado-Morales M., Angelidaki I. (2018). Amino acids production focusing on fermentation technologies–A review. Biotechnol. Adv..

[32] Zhang J., Zhang S., Yang X., Qiu L., Gao B., Li R., Chen J. (2016). Reactive extraction of amino acids mixture in hydrolysate from cottonseed meal with di(2-ethylhexyl) phosphoric acid. J. Chem. Technol. Biotechnol..

[33] Kim Y., Park J., Kim M.-J. (2011). Dynamic Kinetic Resolution of Amines and Amino Acids by Enzyme–Metal Cocatalysis. ChemCatChem.

[34] Miyazawa T. (1999). Enzymatic resolution of amino acids via ester hydrolysis. Amino Acids.

[35] Chen S.-T., Huang W.-H., Wang K.-T. (1994). Resolution of Amino Acids in a Mixture of 2-Methyl-2-propanol/water (19:1) Catalyzed by Alcalase via in Situ Racemization of One Antipode Mediated by Pyridoxal 5-Phosphate. J. Org. Chem..

[36] Wang J., Liu X., Feng X. (2011). Asymmetric strecker reactions. Chem. Rev..

[37] Harada K. (1963). Asymmetric Synthesis of α-Amino-acids by the Strecker Synthesis. Nature.

[38] Kouznetsov V.V., Galvis C.E.P. (2018). Strecker reaction and α-amino nitriles: Recent advances in their chemistry, synthesis, and biological properties. Tetrahedron.

[39] Kunz H., Sager W., Pfrengle W., Schanzenbach D. (1988). Reversal of asymmetric induction in stereoselective strecker synthesis on galactosyl amine as the chiral matrix. Tetrahedron Lett..

[40] Ma D., Tian H., Zou G. (1999). Asymmetric Strecker-Type Reaction of α-Aryl Ketones. Synthesis of (S)-αM4CPG, (S)-MPPG, (S)-AIDA, and (S)-APICA, the Antagonists of Metabotropic Glutamate Receptors. J. Org. Chem..

[41] Ma D., Ding K. (2000). Synthesis of Enantiopure α,α-Disubstituted Amino Acids from the Asymmetric Strecker Reaction Products of Aldehydes. Org. Lett..

[42] Robak M.T., Herbage M.A., Ellman J.A. (2010). Synthesis and Applications of tert-Butanesulfinamide. Chem. Rev..

[43] Chen D., Xu M.-H. (2014). Lewis acid promoted diastereoselective addition of TMSCN and TMSCF3 to isatin-derived N-sulfinyl ketimines: Synthesis of optically active tetrasubstituted 3-aminooxindoles. J. Org. Chem..

[44] Cai X.-H., Xie B. (2014). Recent advances in asymmetric Strecker reactions. Arkivoc.

[45] de Bruin G., Mock E.D., Hoogendoorn S., van den Nieuwendijk A.M., Mazurek J., van der Marel G.A., Florea B.I., Overkleeft H.S. (2016). Enantioselective synthesis of adamantylalanine and carboranylalanine and their incorporation into the proteasome inhibitor bortezomib. Chem. Commun..

[46] Iyer M.S., Gigstad K.M., Namdev N.D., Lipton M. (1996). Asymmetric catalysis of the Strecker amino acid synthesis by a cyclic dipeptide. Amino Acids.

[47] Zuend S.J., Coughlin M.P., Lalonde M.P., Jacobsen E.N. (2009). Scaleable catalytic asymmetric Strecker syntheses of unnatural α-amino acids. Nature.

[48] Sigman M.S., Jacobsen E.N. (1998). Enantioselective Addition of Hydrogen Cyanide to Imines Catalyzed by a Chiral (Salen)Al(III) Complex. J. Am. Chem. Soc..

[49] Kobayashi S., Ishitani H. (2000). Novel binuclear chiral zirconium catalysts used in enantioselective strecker reactions. Chirality Pharmacol. Biol. Chem. Conseq. Mol. Asymmetry.

[50] Sadhukhan A., Saravanan S., Khan N.-u.H., Kureshy R.I., Abdi S.H., Bajaj H.C. (2012). Modified Asymmetric Strecker Reaction of Aldehyde with Secondary Amine: A Protocol for the Synthesis of S-Clopidogrel (An Antiplatelet Agent). J. Org. Chem..

[51] Qiu X.-L., Qing F.-L. (2011). Recent Advances in the Synthesis of Fluorinated Amino Acids. Eur. J. Org. Chem..

[52] Liu Y.-L., Yin X.-P., Zhou J. (2018). Internally Reuse Waste: Catalytic Asymmetric One-Pot Strecker Reaction of Fluoroalkyl Ketones, Anilines and TMSCN by Sequential Catalysis. Chin. J. Chem..

[53] Kawasaki T., Takamatsu N., Aiba S., Tokunaga Y. (2015). Spontaneous formation and amplification of an enantioenriched α-amino nitrile: A chiral precursor for Strecker amino acid synthesis. Chem. Commun..

[54] Miyagawa S., Yoshimura K., Yamazaki Y., Takamatsu N., Kuraishi T., Aiba S., Tokunaga Y., Kawasaki T. (2017). Asymmetric Strecker Reaction Arising from the Molecular Orientation of an Achiral Imine at the Single-Crystal Face: Enantioenriched l-and d-Amino Acids. Angew. Chem. Int. Ed..

[55] Sögütoglu L.-C., Steendam R.R., Meekes H., Vlieg E., Rutjes F.P. (2015). Viedma ripening: A reliable crystallisation method to reach single chirality. Chem. Soc. Rev..

[56] Viedma C. (2005). Chiral symmetry breaking during crystallization: Complete chiral purity induced by nonlinear autocatalysis and recycling. Phys. Rev. Lett..

[57] Baglai I., Leeman M., Wurst K., Kaptein B., Kellogg R.M., Noorduin W.L. (2018). The Strecker reaction coupled to Viedma ripening: A simple route to highly hindered enantiomerically pure amino acids. Chem. Commun..

[58] van der Meijden M.W., Leeman M., Gelens E., Noorduin W.L., Meekes H., van Enckevort W.J.P., Kaptein B., Vlieg E. (2009). Kellogg, R.M. Attrition-Enhanced Deracemization in the Synthesis of Clopidogrel-A Practical Application of a New Discovery. Org. Process. Res. Dev..

[59] Jumbam N.D., Masamba W. (2020). Bio-Catalysis in Multicomponent Reactions. Molecules.

[60] Vongvilai P., Ramström O. (2009). Dynamic Asymmetric Multicomponent Resolution: Lipase-Mediated Amidation of a Double Dynamic Covalent System. J. Am. Chem. Soc..

[61] Chrzanowska M., Grajewska A., Meissner Z., Rozwadowska M., Wiatrowska I. (2012). A concise synthesis of tetrahydroisoquinoline-1-carboxylic acids using a Petasis reaction and Pomeranz–Fritsch–Bobbitt cyclization sequence. Tetrahedron.

[62] Wu P., Givskov M., Nielsen T.E. (2019). Reactivity and Synthetic Applications of Multicomponent Petasis Reactions. Chem. Rev..

[63] Boguszewski P.A., Davies J.W., Marsh P.A., Williamson M. (2008). MEDI 309-Polymer assisted, high throughput methods for Petasis and Ugi reactions. Abstracts of Papers of the American Chemical Society.

[64] Zhang J., Yun F., Xie R., Cheng C., Chen G., Li J., Tang P., Yuan Q. (2016). Petasis three-component reaction accelerated by trifluoroacetic acid: Synthesis of indoline-derived glycines. Tetrahedron Lett..

[65] Frauenlob R., García C., Bradshaw G.A., Burke H.M., Bergin E. (2012). A Copper-Catalyzed Petasis Reaction for the Synthesis of Tertiary Amines and Amino Esters. J. Org. Chem..

[66] Cornier P.G., Delpiccolo C.M.L., Boggián D.B., Mata E.G. (2013). Solid-phase Petasis multicomponent reaction for the generation of β-lactams 3-substituted with non-proteinogenic α-amino acids. Tetrahedron Lett..

[67] Potowski M., Esken R., Brunschweiger A. (2020). Translation of the copper/bipyridine-promoted Petasis reaction to solid phase-coupled DNA for encoded library synthesis. Biorg. Med. Chem..

[68] Petasis N.A., Goodman A., Zavialov I.A. (1997). A new synthesis of α-arylglycines from aryl boronic acids. Tetrahedron.

[69] Liepouri F., Bernasconi G., Petasis N.A. (2015). Component-Selective and Stereocontrolled One-Step Three-Component Reaction among Aldehydes, Amines, and Allenyl Boronic Acids or Allenyl Pinacolboronates. Org. Lett..

[70] Murafuji T., Tasaki Y., Fujinaga M., Tao K., Kamijo S., Ishiguro K. (2017). Blue Amino Acids Derived from Azulen-1-ylboronic Acid Pinacol Ester via the Petasis Reaction. Synthesis.

[71] Petasis N.A., Zavialov I.A. (1997). A New and Practical Synthesis of α-Amino Acids from Alkenyl Boronic Acids. J. Am. Chem. Soc..

[72] Tao C.-Z., Zhang Z.-T., Wu J.-W., Li R.-H., Cao Z.-L. (2014). Synthesis of unnatural N-glycosyl α-amino acids via Petasis reaction. Chin. Chem. Lett..

[73] Naskar D., Roy A., Seibel W.L., Portlock D.E. (2003). Hydroxylamines and sulfinamide as amine components in the Petasis boronic acid–Mannich reaction: Synthesis of N-hydroxy or alkoxy-α-aminocarboxylicacids and N-(tert-butyl sulfinyl)-α-amino carboxylicacids. Tetrahedron Lett..

[74] Nielsen S.D., Smith G.P., Begtrup M., Kristensen J.L. (2011). Synthesis of N-alkylated amino acids using fluorous-tagged hydroxylamines. Tetrahedro.

[75] Dhudshia B., Tiburcio J., Thadani A.N. (2005). Diastereoselective allylation and crotylation of N-unsubstituted imines derived from ketones. Chem. Commun..

[76] Diehl A.M., Ouadoudi O., Andreadou E., Manolikakes G. (2018). Sulfonamides as Amine Component in the Petasis-Borono Mannich Reaction: A Concise Synthesis of α-Aryl-and α-Alkenylglycine Derivatives. Synthesis.

[77] Candeias N.R., Cal P.M.S.D., André V., Duarte M.T., Veiros L.F., Gois P.M.P. (2010). Water as the reaction medium for multicomponent reactions based on boronic acids. Tetrahedron.

[78] Ishiyama T., Matsuda N., Murata M., Ozawa F., Suzuki A., Miyaura N. (1996). Platinum(0)-Catalyzed Diboration of Alkynes with Tetrakis(alkoxo)diborons:  An Efficient and Convenient Approach to cis-Bis(boryl)alkenes. Organometallics.

[79] Sridhar T., Berrée F., Sharma G.V.M., Carboni B. (2014). Regio- and Stereocontrolled Access to γ-Boronated Unsaturated Amino Esters and Derivatives from (Z)-Alkenyl 1,2-Bis(boronates). J. Org. Chem..

[80] Yamaoka Y., Miyabe H., Takemoto Y. (2007). Catalytic Enantioselective Petasis-Type Reaction of Quinolines Catalyzed by a Newly Designed Thiourea Catalyst. J. Am. Chem. Soc..

[81] Lou S., Schaus S.E. (2008). Asymmetric Petasis Reactions Catalyzed by Chiral Biphenols. J. Am. Chem. Soc..

[82] Churches Q.I., Stewart H.E., Cohen S.B., Shröder A., Turner P., Hutton C.A. (2008). Stereoselectivity of the Petasis reaction with various chiral amines and styrenylboronic acids. Pure Appl. Chem..

[83] Churches Q.I., White J.M., Hutton C.A. (2011). Synthesis of β,γ-Dihydroxyhomotyrosines by a Tandem Petasis–Asymmetric Dihydroxylation Approach. Org. Lett..

[84] Li Y., Xu M.-H. (2012). Lewis Acid Promoted Highly Diastereoselective Petasis Borono-Mannich Reaction: Efficient Synthesis of Optically Active β,γ-Unsaturated α-Amino Acids. Org. Lett..

[85] Inokuma T., Suzuki Y., Sakaeda T., Takemoto Y. (2011). Synthesis of Optically Active N-Aryl Amino Acid Derivatives through the Asymmetric Petasis Reaction Catalyzed by a Novel Hydroxy–Thiourea Catalyst. Chem. An Asian J..

[86] Morozova V.A., Beletskaya I.P., Titanyuk I.D. (2017). Synthesis of enantiopure cyclic amino acid derivatives via a sequential diastereoselective Petasis reaction/ring closing olefin metathesis process. Tetrahedron: Asymmetry.

[87] Bułyszko I., Chrzanowska M., Grajewska A. (2015). Rozwadowska, M.D. Synthesis of (+)-6,7-Dimethoxy-1,2,3,4-tetrahydroisoquinoline-1-carboxylic Acid, a Diastereoselective Approach. Eur. J. Org. Chem..

[88] Koolmeister T., Södergren M., Scobie M. (2002). The first example of chiral induction using homochiral boronic esters in the Petasis reaction. Tetrahedron Lett..

[89] Wang Y., Guan R., Sivaguru P., Cong X., Bi X. (2019). Silver-Catalyzed anti-Markovnikov Hydroboration of C–C Multiple Bonds. Org. Lett..

[90] Agahi R., Challinor A.J., Carter N.B., Thomas S.P. (2019). Earth-abundant metal catalysis enabled by counterion activation. Org. Lett..

[91] Jang W.J., Song S.M., Moon J.H., Lee J.Y., Yun J. (2017). Copper-catalyzed enantioselective hydroboration of unactivated 1, 1-disubstituted alkenes. J. Am. Chem. Soc..

[92] Joshi-Pangu A., Ma X., Diane M., Iqbal S., Kribs R.J., Huang R., Wang C.-Y., Biscoe M.R. (2012). Palladium-catalyzed borylation of primary alkyl bromides. J. Org. Chem..

[93] Dudnik A.S., Fu G.C. (2012). Nickel-catalyzed coupling reactions of alkyl electrophiles, including unactivated tertiary halides, to generate carbon–boron bonds. J. Am. Chem. Soc..

[94] Muncipinto G., Moquist P.N., Schreiber S.L., Schaus S.E. (2011). Catalytic Diastereoselective Petasis Reactions. Angew. Chem. Int. Ed..

[95] Gong Y.-C., Wang Y., Li E.-Q., Cui H., Duan Z. (2019). Enantio- and Diastereoselective Synthesis of β-Aryl-β-pyrazolyl α-Amino Acid Esters via Copper-Catalyzed Reaction of Azomethine Ylides with Benzylidenepyrazolones. Adv. Synth. Catal..

[96] Menor-Salván C. (2018). From the dawn of organic chemistry to astrobiology: Urea as a foundational component in the origin of nucleobases and nucleotides. Prebiotic Chemistry and Chemical Evolution of Nucleic Acids.

[97] Hall D.G. (2005). Structure, properties, and preparation of boronic acid derivatives. Overview of their reactions and applications. Boronic Acids.

